# Nondisjunction of a Single Chromosome Leads to Breakage and Activation of DNA Damage Checkpoint in G2

**DOI:** 10.1371/journal.pgen.1002509

**Published:** 2012-02-16

**Authors:** Oliver Quevedo, Jonay García-Luis, Emiliano Matos-Perdomo, Luis Aragón, Félix Machín

**Affiliations:** 1 Unidad de Investigación, Hospital Universitario Nuestra Señora de Candelaria, Santa Cruz de Tenerife, Spain; 2 Cell Cycle Group, MRC Clinical Sciences Centre, Imperial College London, London, United Kingdom; Indiana University, United States of America

## Abstract

The resolution of chromosomes during anaphase is a key step in mitosis. Failure to disjoin chromatids compromises the fidelity of chromosome inheritance and generates aneuploidy and chromosome rearrangements, conditions linked to cancer development. Inactivation of topoisomerase II, condensin, or separase leads to gross chromosome nondisjunction. However, the fate of cells when one or a few chromosomes fail to separate has not been determined. Here, we describe a genetic system to induce mitotic progression in the presence of nondisjunction in yeast chromosome XII right arm (cXIIr), which allows the characterisation of the cellular fate of the progeny. Surprisingly, we find that the execution of karyokinesis and cytokinesis is timely and produces severing of cXIIr on or near the repetitive ribosomal gene array. Consequently, one end of the broken chromatid finishes up in each of the new daughter cells, generating a novel type of one-ended double-strand break. Importantly, both daughter cells enter a new cycle and the damage is not detected until the next G2, when cells arrest in a Rad9-dependent manner. Cytologically, we observed the accumulation of damage foci containing RPA/Rad52 proteins but failed to detect Mre11, indicating that cells attempt to repair both chromosome arms through a MRX-independent recombinational pathway. Finally, we analysed several surviving colonies arising after just one cell cycle with cXIIr nondisjunction. We found that aberrant forms of the chromosome were recovered, especially when *RAD52* was deleted. Our results demonstrate that, in yeast cells, the Rad9-DNA damage checkpoint plays an important role responding to compromised genome integrity caused by mitotic nondisjunction.

## Author Summary

When cells divide they must segregate copies of their chromosomes to each of their daughters. A particular harmful situation arises when those copies are glued to each other (i.e., nondisjunction) at the moment of division. Previously, it has been possible to genetically favour this scenario, yet it has been difficult to limit the extent of nondisjunction to a single chromosome. We have developed and studied a yeast model where we control nondisjunction of one of its sixteen chromosomes. We show that dividing cells manage to complete nuclear and cell fission and therefore break that chromosome. We further show that new daughter cells then trigger a DNA damage response, yet only after they initiate a new round of replication. Remarkably, an uncommon repair strategy seems to be used to deal with this damage, which involves part of the homologous recombination machinery (i.e., RPA complex and Rad52) but lacks its primary sensor Mre11. Importantly though, both daughter cells arrest their cell cycle in G2 to prevent further damage from occurring. After a while, the cell that still carries an entire copy of the chromosome often survives, leading to aberrant forms of the chromosome in the progeny.

## Introduction

Chromosomes lagging or bridging during anaphase are believed to be one of the main sporadic causes of cytokinesis failure, which leads to tetraploid cells with multicentrosomes, a hallmark of early tumourigenesis [1], [2]. Conversely, if these anaphase bridges break apart, chromosomes could enter the so-called breakage-fusion-bridge cycle [3]–[5], which has been related to oncogene amplification and intratumour heterogeneity [6]–[8]. Carcinogens such as cigarette smoke, dysfunction of key cancer genes, bacterial toxins, and, paradoxically, many antitumour chemotherapeutic treatments (e.g. topoisomerase inhibitors) are known to cause anaphase bridges [9]–[12].

Chromosomes bridge in anaphase because they have either more than one centromere or problems in resolving the sister chromatids. Most of our knowledge on the biology of sister chromatid resolution comes from studies in yeast. In *Saccharomyces cerevisiae*, as in the rest of eukaryotes, sister chromatids are kept together after replication by both the cohesin complex and DNA-DNA topological entanglement arising from DNA metabolism (i.e., catenations) [13]. During anaphase onset, cohesion is lost through the regulated cleavage of cohesin by separase [14], and catenations are removed by the combined actions of condensin [15] and type 2 topoisomerase (Top2) [16]. Yeast mutants for any of these players show knotted nuclear masses in anaphase with trailing distal chromosome regions which cannot be resolved in otherwise bipolarly attached centromeres [15]–[20]. Despite these anaphase problems, all these mutants often perform cytokinesis, leading to a “cut” phenotype characterized by aneuploid daughter cells carrying broken chromosomes [14], [17], [21]. Not surprisingly, many daughter cells are not able to enter a new cell cycle after cytokinesis [14]–[16]. This has precluded use of those mutants as tools to follow up the short-term consequences in the progeny of anaphase bridges formed by unresolved sister chromatids.

The last genomic region to get resolved in yeast is the ribosomal DNA array (rDNA) [19], [22]–[24]. Importantly, resolution at this locus depends on a third player, besides condensin and Top2: the late mitotic phosphatase Cdc14 [22]–[27]. This is because Cdc14 inactivates transcription by RNA polymerase I in late anaphase, which allows the loading of condensin to the rDNA, its condensation and further resolution with the help of Top2 [22], [23], [27]–[31]. Other findings also suggest that these Cdc14 actions could serve to finish up replication within this locus [32], [33]. When Cdc14 is inactivated by means of thermosensitive conditional alleles such as *cdc14-1*, the anaphase segregation problem is much milder than that observed for the other aforementioned mutants. Indeed, *cdc14-1* cells get arrested in telophase with the bulk of the nuclear masses segregated yet the rDNA bridging between mother and daughter cells [23], [24].

In a previous report we demonstrated that re-activation of the thermosensitive protein Cdc14-1 restores its cell cycle functions and is enough to exit mitosis [28]. Nevertheless, a portion of cells do this in spite of failing, in the end, to segregate the rDNA. Because little is known about the behaviour and fate of cells that commit to a new cell cycle once they have failed to resolve sister chromatids, we decided to address these questions taking advantage of this *cdc14-1* re-activation phenotype. Herein, we show that *cdc14-1* release leads to severing of the rDNA anaphase bridge and a new Rad9-dependent G2/M arrest. We followed the DNA damage response (DDR) in these cells and observed that they elicit a Rad52 long-lasting response that is independent of Mre11. We further discuss how our system provides a model for the study of DNA double strand breaks (DSB) where the ends finish up in different compartments (i.e., “one-ended”).

## Results

### Release from a *cdc14-1* telophase block leads to a pre-anaphase arrest in the following cell cycle

**Figure 1:**
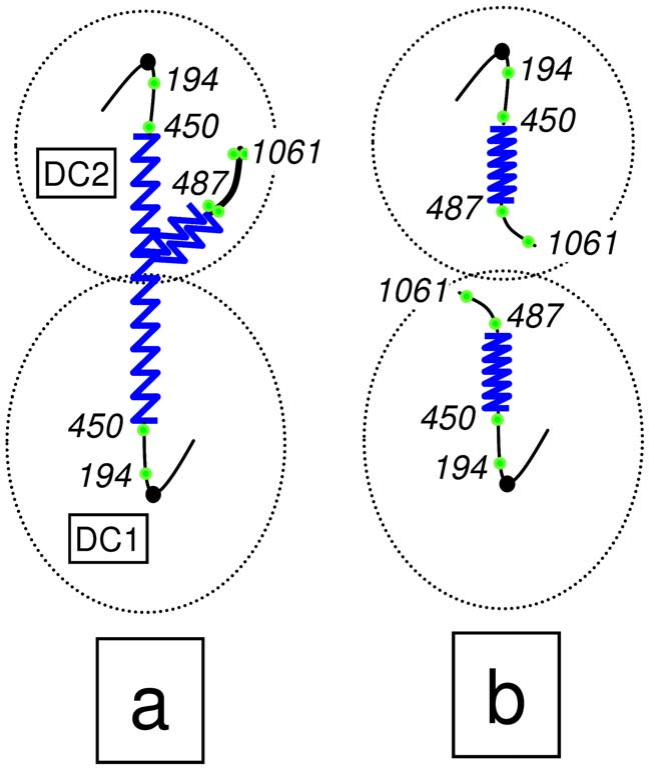
Scheme of the chromosome XII anaphase bridge (cXIIr) in a *cdc14-1* telophase block. According to our previous findings [23], [28], two major phenotypes are found at either the telophase block or after the release (see main text for details): (a) non-disjunction goes from within the rDNA to the telomere of the chromosome XII right arm (cXIIr); and (b) the chromosome is fully segregated. Chromosome positions of *tetOs* used to determine the extent of non-disjunction are numbered and shown as green dots; rDNA is depicted as a serrated blue line; and thicker lines indicate non-resolved sister chromatids. “DC1” depicts the daughter-to-be cell which carries just one copy of the resolved part of chromosome XII (from left telomere to somewhere within the rDNA); whereas “DC2” carries one entire sister chromatid plus the unresolved part of the other one (from somewhere within the rDNA to the right telomere).

Since the pioneering works by Hartwell and collaborators on yeast cell cycle control, it is known that conditional mutants for two essential genes, *CDC14* and *CDC15*, give a telophase block with mostly binucleated dumbbell cells [34]. Nevertheless, we also now know that at least some *cdc14* mutants have problems in the resolution and segregation of chromosomes during anaphase [22]–[24], [33]. As for the rDNA-bearing chromosome XII right arm (cXIIr), the telophase block elicited by the *cdc14-1* allele prevents sister chromatid resolution, and therefore segregation, of regions that extend from somewhere within the large rDNA locus to the end of that chromosome arm [23]. Consequently, cXIIr forms an anaphase bridge between the connected daughter nuclei (Figure 1 for a scheme, “a” phenotype). In a previous work, we surprisingly found that, after reactivating the thermosensitive Cdc14-1 protein, cells were able to resume the cell cycle in spite of often failing to complete the resolution and segregation of such distal regions [28]. In general, around 50% of the cells coming out of a *cdc14-1* block do not change their missegregation pattern, whereas the other 50% fully complete segregation of cXIIr (Figure 1, “a” & “b” phenotypes respectively).

**Figure 2:**
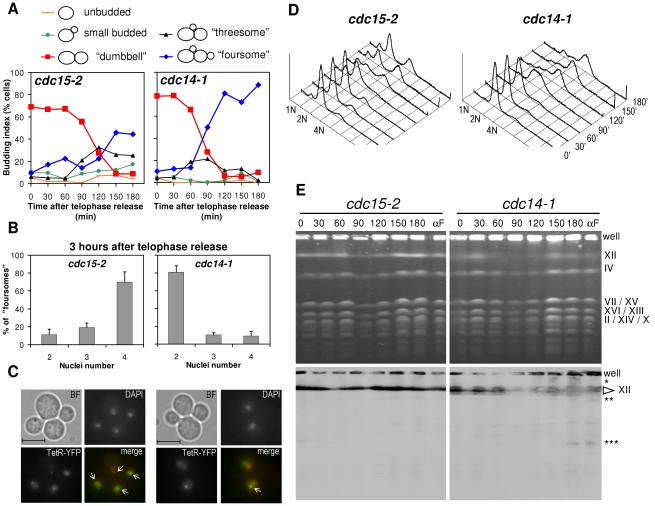
Cells arrest in G2 after a *cdc14-1* release with chromosome XII integrity compromised. Strains FM588 (*cdc15-2 tetO:1061 TetR-YFP*) and FM322 (*cdc14-1 tetO:1061 TetR-YFP*) were arrested in telophase by incubation at 37°C for 3 hours (time = 0 minutes) and then released from the arrest by dropping the temperature to 25°C. Samples were taken every 30 minutes for 3 hours and analysed by microscopy (A, B & C), flow cytometry (D) and PFGE (E). (A) Time course of cell morphology after the release. Note the transition from the telophase arrest (dumbbell cells) to the main foursome category. (B) Number of nuclei in foursomes 3 h after the release. Note how *cdc15-2* has entered a new anaphase (four nuclei) while *cdc14-1* is still stuck in a pre-anaphase stage (2 nuclei). The charts represent mean ± SEM, n = 3 (one of which is the particular experiment used for the rest of the figure). (C) Micrographs of the main foursome types observed for *cdc15-2* and *cdc14-1* at that time point. We used the *tetOs:1061* to assess cXIIr segregation (main text for details). White arrows point to *tetOs*. Bar, 5 µm. (D) Flow cytometry analyses of the releases. Peaks of DNA content (1 N, 2 N & 4 N) are indicated. Telophase arrest gives a 2 N peak. 4 N peak appears in foursomes provided each daughter replicates its DNA. Note how the 4 N peak is reached after the *cdc14-1* release. (E) Pulsed-field gel electrophoresis of the same releases. Note that: (i) most chromosomes were replicated after the *cdc14-1* release (chromosome bands faded away at 90′ and reappeared by 150′); (ii) chromosome XII band specifically faded away from time 30′ in *cdc14-1* and never reappeared fully; and (iii) lower (single-asterisk) and faster (double- and triple-asterisks) migrating forms of chromosome XII appeared after the *cdc14-1* release. Lanes for samples taken from a release into a new G1 block (αF) are also included. Correspondence between main bands and chromosomes is indicated on the right.

We began our study by closely monitoring the cell cycle that follows *cdc14-1* release in a strain where the cXIIr telomere is labelled (*tetO:1061*). We further included a side-by-side isogenic *cdc15-2* strain as a control, after confirming that the cXIIr is fully segregated in its telophase block (Figure S1). In the same experiment we monitored: (i) the budding pattern after the release (Figure 2A); (ii) the morphological changes of the nuclei and the overall resolution and segregation of the cXIIr telomere (Figure 2B and 2C); (iii) the changes in DNA content by flow cytometry (i.e., bulk replication) (Figure 2D); and (iv) chromosome behaviour in a pulsed-field gel electrophoresis (PFGE) (i.e., individual chromosome replication and integrity) (Figure 2E). For the first two, we looked under the microscope and counted individual cells. The telophase release led to rebudding of the initial dumbbell mitotic cell for both mutants (Figure 2A). Virtually all cells were able to resume the cell cycle in a synchronous way as indicated by the drop of the dumbbell category (in red) to values below 10%. Around 120 minutes after the release, most cells had rebudded again. Since most daughter cells remain together for a while after the release, this rebudding gave “threesomes” (i.e., single-rebudded or three cell bodies) and “foursomes” (i.e., double-rebudded or four cell bodies). Foursomes (in blue) remained the most abundant category from 120 minutes onwards (∼90% in the *cdc14-1* release, and ∼50% in the *cdc15-2* release). The lesser amount of foursomes in the *cdc15-2* release occurred because daughter cells from this mutant were eventually able to separate from each other, whereas daughters for *cdc14-1* remained tightly together even long after becoming foursomes (see below). When we followed the release for longer periods we noticed that the budding pattern of the *cdc15-2* release became complex and tended to be oscillatory. By contrast, *cdc14-1* was much simpler and many cells stalled as the foursome category throughout (data not shown and Figure S2). A critical difference between the observed threesomes and foursomes for *cdc15-2* and *cdc14-1* became evident when we looked at the nuclei by DAPI. Thus, *cdc15-2* foursomes had 4 nuclei (i.e., both daughters have entered and completed another nuclear division round) in ∼70% of the cases at minute 180. By contrast, the *cdc14-1* release had less than 10% of the foursomes in this situation at that time (Figure 2B and Figure S2). When we looked at the segregation pattern of the cXIIr telomere (*tetO:1061*) in these strains, we found that *cdc15-2* always segregated it faithfully in the two cell division that took place (94.0±0.1% [mean ± SEM, n = 3] of foursomes with four nuclei had one *tetO* in each nucleus) (Figure 2C for a representative micrograph). In the case of *cdc14-1*, 63.6±1.7% (mean ± SEM, n = 3) of foursomes with 2 nuclei had already missegregated cXIIr as expected [28], and no more cell divisions proceeded in that period (Figure 2C).

We repeated this block-and-release experiment in different yeast strains and backgrounds and found that the main conclusion was conserved (i.e., long-lasting arrest of many *cdc14-1* cells as foursomes in a pre-anaphase stage). However, we observed slight differences in terms of synchrony after the release, time of rebudding, and number of daughter cells able to separate from each other. For instance, in the W303 background, *cdc15-2* cells got released earlier and the synchrony was much better throughout (Figure S2).

We further explored the spindle apparatus (spindle itself using Tub1-GFP and spindle pole bodies with Tub4-CFP) in the *cdc14-1* foursomes and observed that each of the two nuclei contained duplicated spindle pole bodies and a metaphase-like spindle (Figure S3). This shows that the single nucleus observed for each daughter cell in the *cdc14-1* foursome represents a genuine pre-anaphase arrest and not a highly tangled anaphase.

### Daughter cells complete DNA replication after a *cdc14-1* release

The fact that *cdc14-1* cells stalled as binucleated foursomes after the telophase release indicated that cells got arrested somewhere between S phase (whose beginning coincides with the rebudding event) and anaphase. We next narrowed the window of this arrest to G2/M by demonstrating that cells completed DNA replication after the *cdc14-1* release. This was possible because, at the time we took samples for microscopy in the above-mentioned experiment, we also took samples for following DNA replication in the cell population by flow cytometry and PFGE (Figure 2D and 2E).

When we performed flow cytometry analysis, we observed a duplication of the DNA amount in cells coming from a *cdc14-1* release (Figure 2D). Since these cells ended up as foursomes, replication could be clearly assessed by simply observing how cells transited from a 2 N to a 4 N peak. In the case of *cdc15-2*, the assessment was a little more difficult since the release gave rise to a complex mixture of single cells (both unbudded and budded) and rebudded cells still connected through the cell wall (threesomes and foursomes). However, the three major peaks for DNA content visible during this release accounted well for the observed amounts of each cell type (Figure 2D, left panel), and indicate that these cells also replicated their DNA. An important conclusion we reached from these data is that replication started and finished at the same time for both mutants, at least for the bulk of their DNA.

When we performed PFGE for those samples, we further confirmed that chromosome replication is mostly completed for all chromosomes after the *cdc14-1* release. We ascertained this using the fact that yeast chromosomes cannot enter a PFGE while being replicated [35]. Thus, we observed that a new replication round for all chromosomes started at around minute 90 in both mutants and that most chromosomes re-entered the gel ∼60 minutes later (Figure 2E, upper panel; and S4). This individual chromosome replication behaviour fits well with the bulk replication seen by flow cytometry in Figure 2D.

### Chromosome XII integrity is compromised after a *cdc14-1* release

Although chromosome XII also started replication after the *cdc14-1* release, the recovery of the whole band was incomplete. In fact, we observed just by ethidium bromide staining that chromosome XII became fainter than any other chromosome after the *cdc14-1* release (Figure 2E, upper panels; and S4). This did not happen during the *cdc15-2* release. Importantly, when we performed a southern blot with a probe against the rDNA we could see that other shorter bands appeared (Figure 2E, lower panels, double- and triple-asterisks). These new bands were visible after the new round of replication was completed, but they were also visible if we prevented replication after the *cdc14-1* release by blocking daughter cells in G1. Again, this G1 block also led to a 50% drop of chromosome XII band intensity in the ethidium bromide staining; and this drop was specific to the *cdc14-1* release (Figure S4). Besides, a smear above the band for the entire chromosome was also seen during the *cdc14-1* release, especially after chromosome replication (Figure 2E, lower panels, single-asterisk). Although we do not know what this smear might be, we speculate that it could account for chromosome XII with replication or recombination intermediates. Interestingly, the cell population in this *cdc14-1* strain may have up to three rDNA sizes (Figure 2E, lower panel, cXII arrow); which would indicate that the rDNA array is more unstable in this mutant.

### The new arrest is long-lasting for those cells that were unable to resolve and segregate the chromosome XII right arm

Because the *tetO:1061* can still be segregated in ∼50% of the cells coming from a *cdc14-1* release [28], we next decided to specifically assess whether these cells eventually bypass the arrest as foursomes. For that purpose we filmed any re-budding beyond that point and compared it to the previous cXIIr segregation outcome. We followed up 22 dumbbell *cdc14-1* cells as they transited out of the telophase arrest on minimal medium agarose patches. Twelve out of these 22 cells ended up missegregating the *tetO* (54.5%). As expected, all daughter cells rebudded again, although it took around an hour longer than when we performed the release in liquid cultures (half-life of the dumbbell phenotype was ∼135 minutes for agarose patches versus ∼75 minutes for cells in culture). Importantly, no foursomes that originally missegregated the cXIIr had rebudded a third time by 6 hours after the telophase release (n = 12); whereas 80% of foursomes had done so when cXIIr segregation had been correct (n = 10). This difference is statistically very significant (p<0.001, Fisher's exact test on the 2×2 contingency table).

### Cells complete karyokinesis and cytokinesis after the *cdc14-1* release, even in those cells that missegregated the chromosome XII right arm

At the telophase block, *cdc14-1* strains have daughter-to-be cells still connected through the bud neck as cytokinesis has not yet been completed [36]. A key question to understand the observed G2/M block is to address the fate of the cXIIr anaphase bridge after the release; importantly, whether or not *cdc14-1* cells complete cytokinesis and hence sever the bridge. We addressed this question two ways.

**Figure 3:**
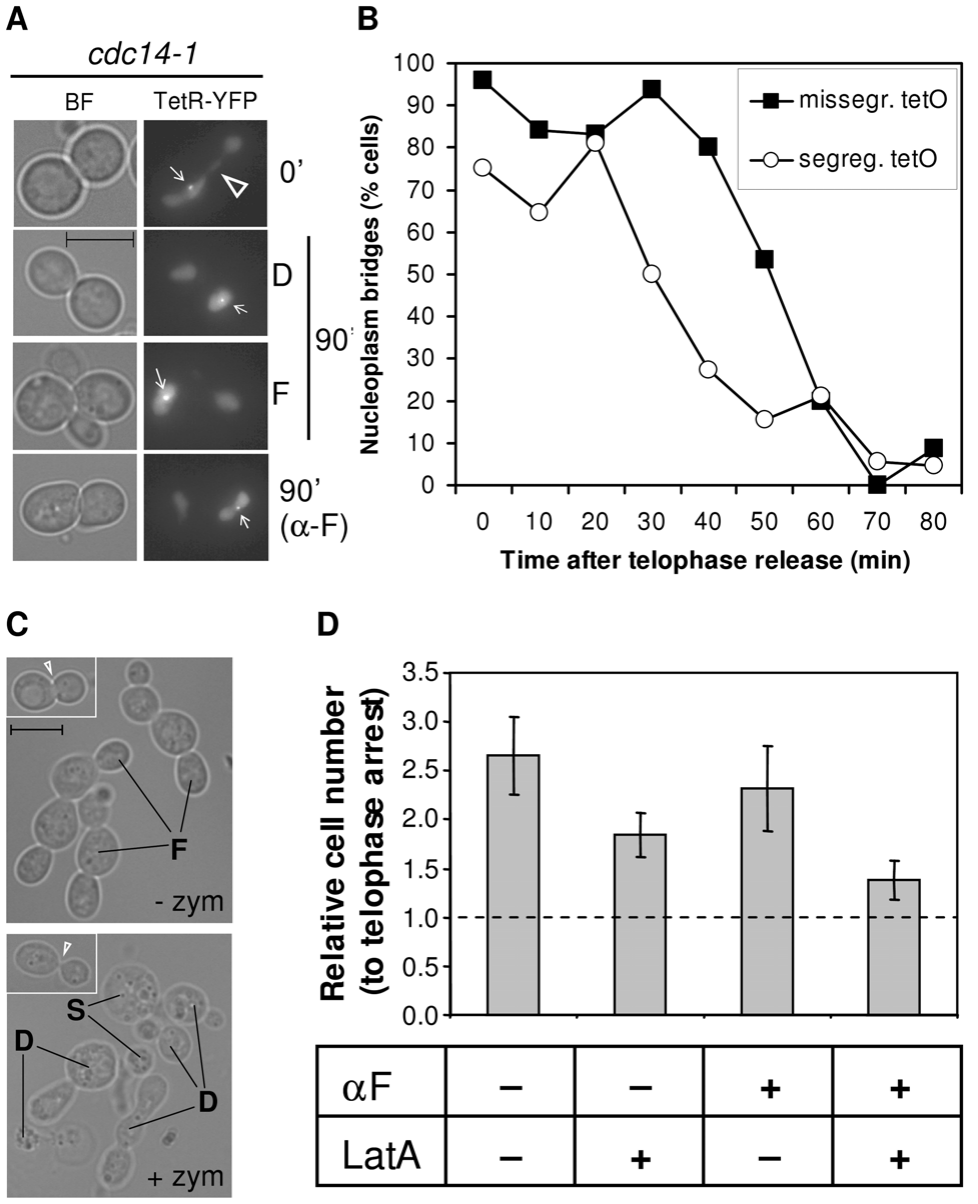
Cells complete karyo- and cytokinesis after a *cdc14-1* release, irrespective of the cXIIr segregation status. (A) Strain FM518 (*cdc14-1 tetO:487 TetR-YFP*) was first arrested at 37°C for 3 hours and micrographed in the conditions described to see the tetR-YFP nucleoplasm bridge (0′, see also Figure S5). Then it was released at 25°C. Part of the yeast culture was released into fresh medium containing alpha-factor to arrest the daughter cells in G1. At 90 minutes after the release, more photos were taken and representative cells are shown. The two main cell morphologies at that time for a normal release, dumbbells [D] and foursomes [F], are depicted. The hollow triangle points to the nucleoplasm bridge. White pointers indicate the missegregated *tetO*. Note that cells have no nucleoplasm bridge. (B) Time course of nucleoplasm bridge disappearance for the same strain relative to the cXIIr segregation status. (C) Strain FM515 (*cdc14-1 RAD52-YFP*) was arrested in telophase by incubation at 37°C for 3 hours. Then the cell culture was shifted back to 25°C to enter a new cell cycle. At the time of the telophase block and 2 hours after the release, samples were taken and fixed with formaldehyde. Contrasted bright field micrographs of representative cells before and after zymolyase treatment are shown. Left corner photos show cells at the telophase block. The white triangle highlights the difference in bud neck thickness after zymolyase treatment. Main photos depict cells 2 hours after the telophase release. “F” points to foursomes, “D” to dumbbells and “S” to single cells. No foursomes (<3%) were seen after zymolyase treatment. (D) Strain FM515 (*cdc14-1 RAD52-YFP*) was treated to inhibit cytokinesis (+LatA) and/or cell cycle progression beyond G1 (+αF). The number of cells was counted in a haemocytometer after the zymolyase treatment. The chart represents cell number two hours after the release relative to their telophase block (mean ± SEM, n = 3).

First we looked at karyokinesis microscopically (i.e., nuclear fission) in a strain where the distal part of the rDNA is tagged (*tetO:487*) and the nuclear TetR-YFP is overexpressed. In this strain we can see both the cXIIr bridge and the nucleoplasm. When Z stacks of microscope pictures were taken at the *cdc14-1* block, the *tetO:487* was seldom segregated and a clear nucleoplasm bridge was visible across the bud neck (Figure 3A, 0′ picture; Figure S5, hollow pointers). The nucleoplasm bridge (soluble TetR-YFP, do not mistake nucleoplasm bridge for anaphase bridge) was also observed for all cells blocked with the *cdc15-2* allele. This suggests that karyokinesis has not yet taken place in both telophase blocks. Noticeably, the nucleoplasm bridge had bulges in the *cdc14-1* block (Figure S5, filled pointers), yet was a thin and straight line in the *cdc15-2* block. We tested whether this bulge accommodates the unresolved rDNA and distal regions of the cXIIr by using another yeast strain that also carries the nucleolar marker Net1 fused to CFP. We found that the nucleolus colocalized with the bulge in more than 95% of the cells (Figure S5B). After the *cdc14-1* telophase release, the nucleoplasm bridge eventually disappeared in dumbbell cells and was never visible in daughter cells that had already rebudded (Figure 3A, dumbbells [D] and foursomes [F] at minute 90). A similar behaviour was seen for *cdc15-2* release (data not shown). Importantly, no *cdc14-1* foursomes had a nucleoplasm bridge, even if they had previously missegregated cXIIr. A conclusive proof that karyokinesis took place before the daughter cells became foursomes was obtained when cells were arrested in G1 right after the *cdc14-1* release. Thus, the nucleoplasm bridge was never visible after the release in cells treated with alpha-factor, irrespective of the cXIIr segregation status (Figure 3A, photo α-F at minute 90).

Although rDNA-missegregating cells ended up performing karyokinesis, it was somehow striking we did not observe a delay in the cell cycle during the *cdc14-1* release. Such delay is expected since a checkpoint has been described to sense the presence of anaphase bridges in yeast (i.e., NoCut checkpoint) [37], [38]. We took advantage of having the aforementioned strain to look at the nucleoplasm and the cXIIr bridges simultaneously and check whether the maintenance of the cXIIr bridge after the release correlated with a delay in the karyokinesis (Figure 3B). We performed a time course of the *cdc14-1* release and followed daughter cells (as dumbbells) throughout the new G1. We observed that the nucleoplasm bridge (again do not mistake for the cXIIr bridge) took around 20 minutes longer to be severed in those cells that finally failed to segregate the cXIIr (Figure 3B, half-life for the nucleoplasm bridge was ∼30 minutes for cells with segregated cXIIr versus ∼50 minutes for missegregated cXIIr). We believe that this 20 minute delay in karyokinesis may account for the NoCut checkpoint. In any case, the time of disappearance of the nucleoplasm bridge (i.e., karyokinesis) was short and the NoCut checkpoint did not preclude cells with the cXIIr bridge from finally completing karyokinesis.

In order to confirm that the fate of the cXIIr bridge is to be severed after the *cdc14-1* release, we also looked at cytokinesis indirectly. We employed an assay based on the fact that formaldehyde-fixed cells that have not completed cytokinesis are resistant to separation by cell wall digesting enzymes (i.e., zymolyase) [39]. At the *cdc14-1* block, when most cells were dumbbells, zymolyase treatment was not able to separate the daughters, although the bud neck that connects them became very thin (Figure 3C, left-corner photos). By contrast, foursomes seen two hours after the release could be split in two (Figure 3C, main lower photo). The drop of foursomes after zymolyase treatment was high (from ∼70% to ∼3%). In another set of experiments, we also counted cell number after zymolyase treatment under different chemical conditions to inhibit either cytokinesis or S-phase. To inhibit cytokinesis, we added the F-actin inhibitor Latrunculin A (LatA) [40]. Since its action against cytokinesis is optimal if cells are incubated before they reach telophase but after they have budded, we employed an initial arrest in G2/M [40]. Then we let them transit from the G2/M arrest to the telophase arrest. To inhibit the new S-phase, we blocked cells in G1 with alpha-factor, added at the telophase release. As expected, overall cell number doubled two hours after the release relative to the telophase arrest in a culture without LatA (Figure 3D). Importantly, this separation could be partially prevented by incubating the *cdc14-1* cells with LatA (*cdc14-1* release with versus without LatA gave a p = 0.036, Student's T test). Moreover, we also demonstrated that cytokinesis occurred before the daughters entered the new S-phase. Thus, alpha-factor prevented dumbbells from becoming foursomes after the release, but it did not circumvent cell separation after zymolyase treatment (Figure 3D). Again, this separation was prevented when LatA was added during the G2/M to telophase transition (*cdc14-1* release to alpha-factor with versus without LatA gave a p = 0.028, Student's T test).

On the whole, we can conclude from this set of experiments about chromosome XII integrity (Figure 2E) and karyo/cytokinesis (Figure 3) that cells physically separate from each other irrespective of the presence of the cXIIr bridge. The logical consequence of this should be the generation of at least a DSB near or within the rDNA.

### The new G2/M arrest that follows the *cdc14-1* release is dependent on Rad9

**Figure 4:**
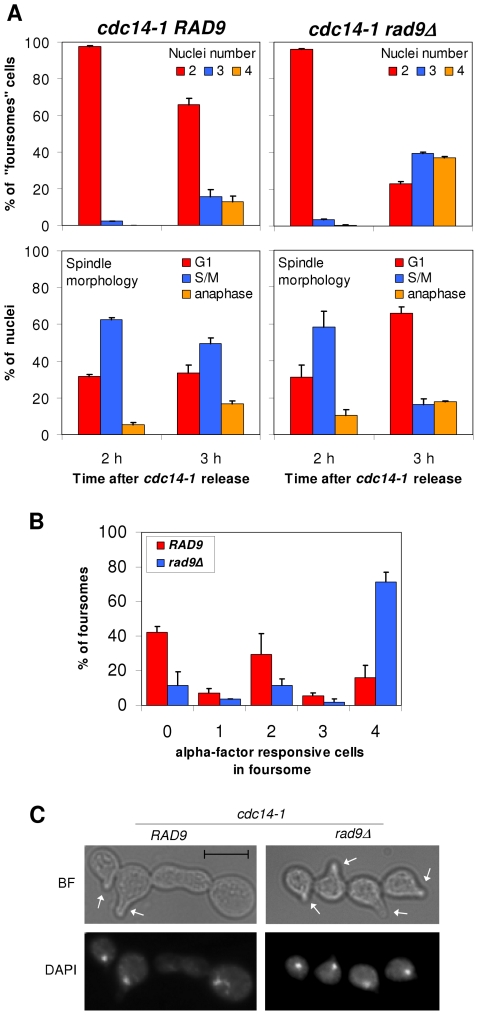
The G2/M arrest that follows a *cdc14-1* release is dependent on Rad9. (A) Strains FM459 (*cdc14-1 TUB1-GFP*) and FM576 (*cdc14-1 rad9Δ TUB1-GFP*) were arrested at 37°C for 3 h and then released. Samples were taken and micrographed 2 and 3 hours after the release. Upper panels show nuclei number after DAPI staining for the major foursome category. Lower panels indicate spindle morphologies for each nucleus in the foursomes (mean ± SEM, n = 3). (B) Strains FM515 (*cdc14-1 RAD52-YFP*) and FM883 (*cdc14-1 rad9Δ RAD52-YFP*) were arrested and released as in A. Two hours after the release, alpha-factor was added and cells were then incubated for 3 more hours before samples were taken and micrographed. Chart represents how many cells in each foursome responded to alpha-factor. Note how *cdc14-1 RAD9* was distributed in three major categories peaking at 0, 2 and 4 responsive cells; whereas most *cdc14-1 rad9Δ* foursomes had all cells responding to alpha-factor (i.e., all progeny passed the G2/M arrest) (mean ± SEM, n = 2). (C) Representative cells of a *cdc14-1 RAD9* foursome with two cells responding to alpha-factor and a *cdc14-1 rad9Δ* foursome with all its 4 cells responding (white arrows point to the shmoo). Note how there are 3 nuclei in the former (two of them in each of the responding cells) and 4 nuclei in the latter (see main text for more details).

The observed karyokinesis, cytokinesis and cXIIr breakage, followed by the arrest in G2/M in the new cell cycle, likely implies that a DSB-mediated DNA damage checkpoint is activated after the *cdc14-1* release. A critical component of this checkpoint is Rad9. Mutants for this protein allow cells with DSBs to enter a new segregation round [41]. Hence, we decided to check whether our observed pre-anaphase arrest could be overcome by deleting *RAD9*. We did this in our *cdc14-1 TUB1-GFP* strain to follow spindle morphology as well as nuclear division after the release (Figure 4A). In contrast to the single mutant *cdc14-1 TUB1-GFP*, the double mutant *cdc14-1 rad9Δ TUB1-GFP* could enter anaphase by 3 hours after the release, becoming foursomes with more than two nuclei masses (i.e., at least one of the daughter cells entered a new anaphase) (Figure 4A, upper panels). Accordingly, when we looked at spindle morphology using Tub1-GFP, we observed a transition from metaphase-like spindles to other patterns in the *cdc14-1 rad9Δ* double mutant (mainly G1-like Tub1 dot signals) (Figure 4A, lower panels).

Besides this, we tested the responsiveness of foursomes to the G1-specific pheromone alpha-factor. We reasoned that if cells were able to progress beyond the G2/M arrest, they would become responsive to the pheromone and change their morphology accordingly (i.e., acquire the shmoo phenotype). Thus, we treated *cdc14-1* cells with the pheromone, not at the time of the release as in other experiments above, but after they became foursomes (2 hours after the release). Then, we left them in alpha-factor for another 3 hours. We first noticed that some *rad9Δ* backgrounds, like the one that carries the *TUB1-GFP*, were able to split the foursomes after this 5 hours incubation time. Therefore, we used one of the W303 backgrounds that kept the foursome category in these conditions. Importantly, all cells in most *cdc14-1 rad9Δ* foursomes were responsive to the pheromone (Figure 4B). Also, these foursomes had four segregated nuclei (Figure 4C). Interestingly, cells in the *cdc14-1 RAD9* foursome distributed in three peaks: one with no responsive cells, one with all four cells responsive, and a third subgroup with just two cells responding to the pheromone (Figure 4B). Within this subgroup, the two responsive cells were always a partner and each has a nucleus (Figure 4C). In order to determine where these two cells come from, we repeated this assay with the *cdc14-1* strain that carries the labelled cXIIr telomere. We found that in 92% of foursomes (n = 39) both responsive cells had a *tetO*, whereas there was no *tetO* in either of the two non-responsive cells. This means that: (i) this subgroup came from *cdc14-1* cells where missegregation occurred in the first place, and (ii) the cell that retained the intact chromosome XII plus the broken cXIIr was able to eventually pass the G2/M arrest.

Therefore, we concluded that cells coming from a *cdc14-1* release activated the Rad9 checkpoint to prevent daughter cells from entering anaphase, and that this G2/M arrest persists for a long time in the daughter cell that lost an intact copy of chromosome XII (i.e., DC1).

### Missegregation of chromosome XII right arm leads to accumulation of Rad52 foci after a *cdc14-1* release

**Figure 5:**
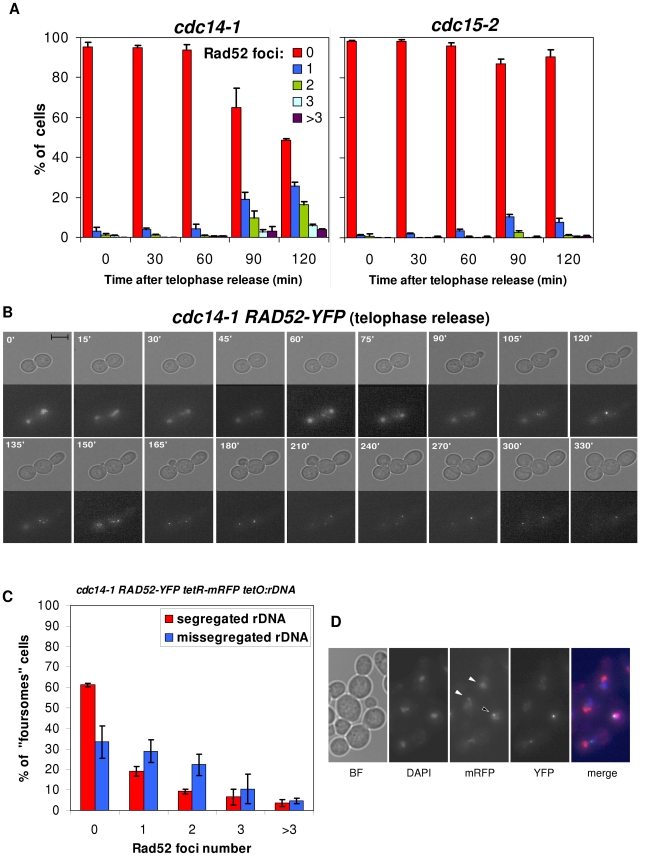
Cells coming from a *cdc14-1* release frequently form Rad52 repair factories, which accumulate when rDNA missegregation had previously occurred. (A) Strains FM531 (*cdc15-2 RAD52-YFP*) and FM515 (*cdc14-1 RAD52-YFP*) were treated as in Figure 2. Cells from samples taken every 30′ were scored (>200 cells each) for number of Rad52-YFP foci (mean ± SEM, n = 3). (B) Time-lapse fluorescence microscopy (every 15–30′ for 6 h) of a FM515 (*cdc14-1 RAD52-YFP*) cell starting at the time of the telophase release. (C) Strain FM551 (*cdc14-1 RAD52-YFP tetO:rDNA tetR-mRFP*) was first arrested in the *cdc14-1* block and then released into a new cell cycle. After 2 hours, Rad52 foci were scored for those foursomes that have either segregated or missegregated the rDNA (mean ± SEM, n = 3). (D) A representative micrograph of two foursomes, one showing segregated *tetOs* (white triangles) and the other one with unresolved *tetOs* (the black triangle). Note the Rad52 focus near the unresolved *tetOs*. In the merged micrograph, mRFP is pseudocoloured in blue and DAPI in red.

The Rad9-dependent cell cycle arrest means that daughter cells sense the DSB(s). Therefore, they must accordingly trigger a DNA damage response (DDR). At this point, we started looking at proteins that cytologically mark this DDR by appearance in nuclear foci. Rad52 is a key mediator in the DDR that comprises the preferred homologous recombination (HR) pathway for repair [42]. This pathway is central in the DDRs that occur throughout S-phase and well into mitosis [43]. We reasoned that, because daughter cells reached and completed S-phase on schedule after a *cdc14-1* release (Figure 2) and then get arrested in G2/M in a Rad9-dependent manner (Figure 2 and Figure 4), Rad52 should be involved in the DDR. Importantly, Rad52-YFP forms widely studied nuclear foci after induced DNA damage [43]. Thus, we looked at Rad52 foci in our telophase block-and-release experiments. We indeed observed foci after a *cdc14-1* release for a subset of cells (Figure 5). Rad52 foci number and intensity were clearly superior in the *cdc14-1* release relative to a side-by-side experiment with the *cdc15-2* strain (Figure 5A). Importantly, there was no difference between the strains when growing asynchronously at 25°C (only ∼5% of budded cells had foci). Foci were observed at the telophase block for neither *cdc14-1* nor *cdc15-2*, further indicating that DNA damage has not yet taken place at this stage. Foci started around 90 minutes after the release and always after rebudding (Figure 5B for a typical time-lapse movie). Furthermore, Rad52 foci were rather dynamic at the beginning of the new S-phase and, within the subgroup that, at some point, had Rad52-YFP foci, tended to end up as either just one major focus in the foursomes (in one of its two nuclei) or 2 foci, one located in each nucleus (Figure 5A and 5B).

Remarkably, the percentage of foursomes acquiring at least one long-lasting single Rad52-YFP focus during the release was ∼50% (Figure 5A). We noticed that this percentage of cells was equivalent to that of rDNA/cXIIr missegregation [28]. We therefore hypothesised that cells with Rad52 foci may represent those that had failed in rDNA segregation. We addressed this important question in two ways. First we made use of a second mutation that worsens rDNA segregation after a *cdc14-1* release (i.e., deletion of *FOB1* gene) [28] (Figure S6). Second, we double labelled two *cdc14-1* strains (one in the S288C background and the other one in W303) with a tag for the rDNA and a tag for the Rad52 protein. For the S288C background, we employed our strain with the *tetO:487* and added a RAD52-RedStar2 allele (Figure S7). As for W303, we employed a previously described strain that bears both Rad52-YFP and a tag inserted within the rDNA (*tetOs*/TetR-mRFP system) [44] and that we made *cdc14-1* (Figure 5C). By using the *cdc14-1 fob1Δ* double mutant, we could correlate worsening of the rDNA segregation with a higher frequency of foursomes carrying at least one bright Rad52 focus (Figure S6; p<0.0001, Pearson's chi-square test). On the other hand, double labelling of Rad52 and the rDNA further and strongly confirmed that Rad52 foci are more frequent in cells that missegregated the rDNA (Figure 5C and Figure S7; p<0.0001, Pearson's chi-square test). Moreover, these strains allowed us to determine that the first and strongest Rad52 focus appeared in the daughter cell that does not carry the *tetOs* (i.e., cell DC1). Thus, 75% of these foci were located in that cell versus only 8% of Rad52 single foci seen in the daughter cell that carries the *tetOs* (the remaining 17% of foursomes had one Rad52 focus in each daughter cell). We believe that this is an important result since the genetic material that each daughter carries in the anaphase bridge is different as stated above (Figure 1, “a” phenotype). Hence, “DC1” cell (the one without the *tetOs*) bears just one broken copy of the resolved part of chromosome XII (from left telomere to somewhere within the rDNA), whereas “DC2” cell carries one entire sister chromatid plus the unresolved part of the other one (from somewhere within the rDNA to the right telomere). The fact that Rad52 foci are stronger and long-lasting in DC1 might indicate that this cell struggles to repair the DSB, while DC2 might end up repairing its broken end. This is in agreement with what we observed when deleted *RAD9* (Figure 4B). Finally, it is worth mentioning that, for those Rad52 foci visible in the *tetO*-carrying nuclei, the fluorescent dots were almost always in close proximity (Figure 5D). However, these Rad52 foci did not localize within the nucleolus when we used a nucleolar marker (Figure S8). This is not surprising though as broken rDNA sequences are transported out of the nucleolus towards nuclear Rad52 factories [44].

Overall, these observations fit well with the prediction of a DDR occurring preferentially in those daughter cells that failed in rDNA segregation during the preceding division.

### Release from the *cdc14-1* block also leads to Rfa1 foci, yet only after cells reach the new S-phase

**Figure 6:**
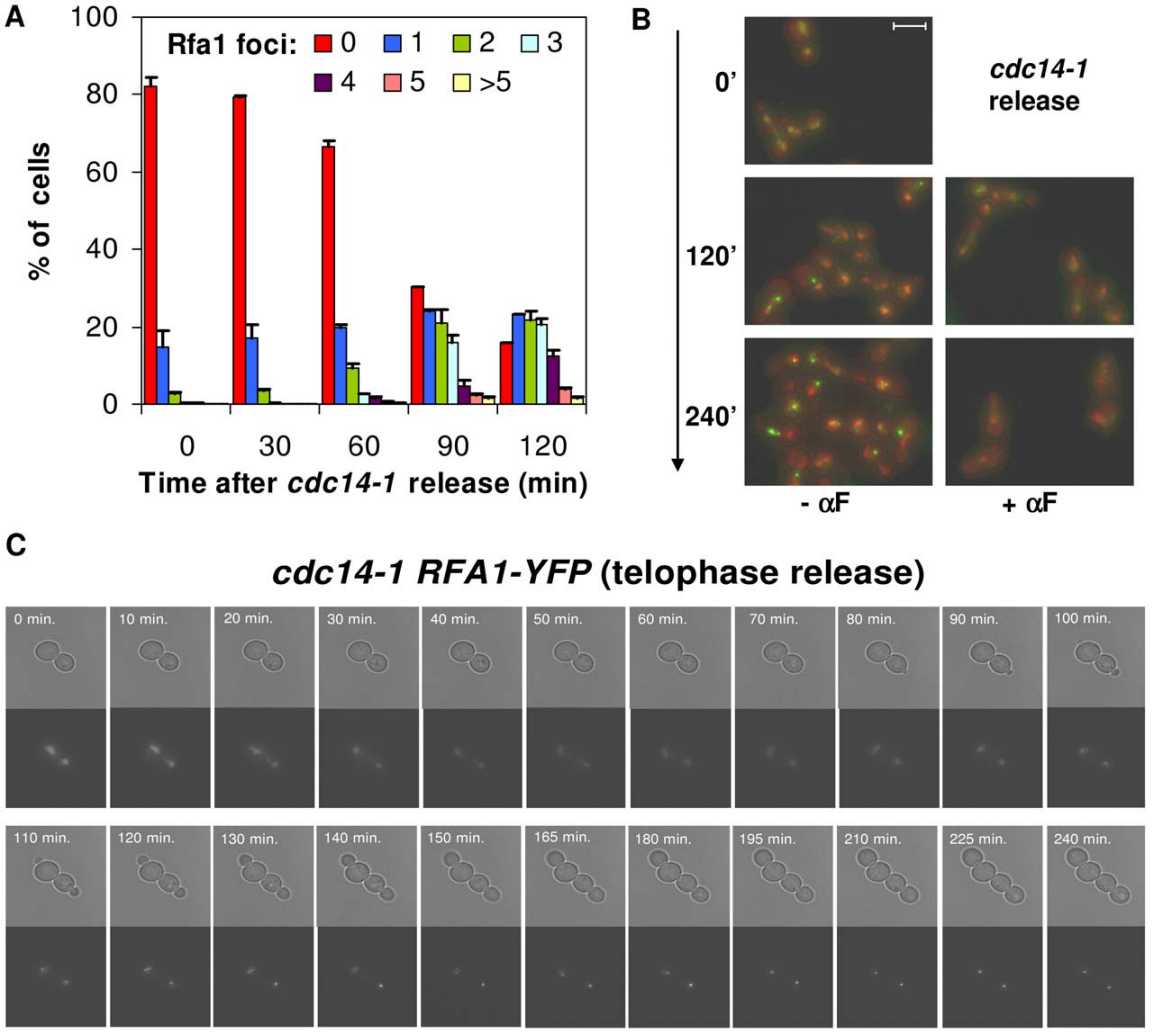
Cells coming from a *cdc14-1* release form Rfa1 factories only after reaching S-phase. (A) Strain FM513 (*cdc14-1 RFA1-YFP*) was treated as in Figure 2. Samples were taken every 30′ after the release and Rfa1-YFP foci quantified per cell (mean ± SEM, n = 3). (B) Representative micrographs of FM513 cells at the telophase arrest (0′), 2 hours (120′) and 4 hours (240′) after the release into fresh medium with or without α-factor. Bright field photos are superimposed over the two fluorescent channels (red for DAPI and green for Rfa1-YFP). (C) Time-lapse fluorescence microscopy (every ∼10–15′ for 4 hours) of one FM513 cell starting at the time of the telophase release.

We expected the DSB to occur shortly after the release into G1, when karyo- and cytokinesis took place (Figure 3) and chromosome XII appeared partly broken (Figure 2E). We were intrigued by the fact that cells did not, however, delay G1 (Figure 2). A possible explanation for this anomaly would be that the DSB is clean (i.e., with little associated single-stranded DNA [ssDNA] at the edges). This is the type of DSB generated by inducible endonucleases like HO as opposed to DSBs obtained after ionizing irradiation, which are rich in ssDNA (i.e., ragged ends) [45], [46]. It has been shown that clean ends are poorly resected in G1, forming little ssDNA, whereas ragged DSBs can already bind ssDNA-binding proteins such as the RPA complex. The formation of ssDNA and the binding of the RPA complex to it are key steps in checkpoint activation [47], [48]. Because of this, we also included the RPA complex as a reporter to study the DDR that follows the *cdc14-1* release. YFP-tagged Rfa1 (one of the complex subunits) also forms foci under the presence of DSBs [49]. Crucially, Rfa1 can form foci in G1 provided that the DSB takes place at this stage and, as just mentioned, the break is ragged [46], [49], [50]. Thus, we observed Rfa1 foci for a subset of cells coming from a *cdc14-1* release (Figure 6A and 6B). Rfa1 foci were as dynamic as those of Rad52 and also tended to end up as a major focus, one per nucleus in the foursome at the most (Figure 6C). Moreover, it was noticeable that Rfa1-YFP eventually gave very intense foci (Figure 6B). Nevertheless, all foci began to appear around 60′–90′ after the release (Figure 6A), and always after rebudding (Figure 6C). Accordingly, foci were not observed in *cdc14-1* cells transiting from telophase to a G1 arrest with alpha-factor (Figure 6B). This likely means that the new type of DSB generated by the severing of the cXIIr bridge has little associated ssDNA (i.e., the DSB is clean) and therefore it is not recognized by the RPA complex in G1.

### The Rad52 response to the severing of the chromosome XII right arm anaphase bridge is independent of Mre11

**Figure 7:**
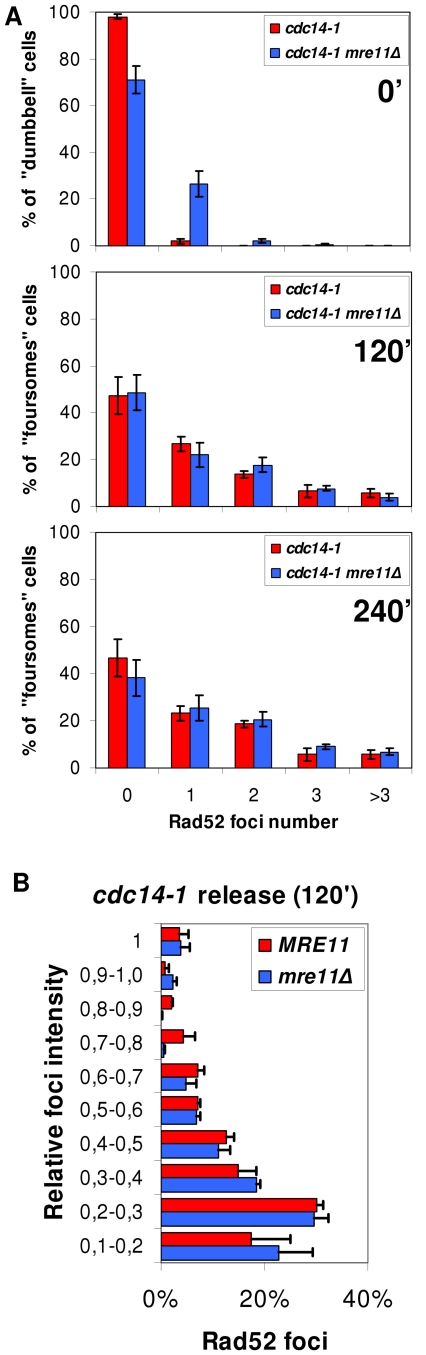
Accumulation of Rad52 foci after the *cdc14-1* release is independent on Mre11. (A) Strains FM515 (*cdc14-1 RAD52-YFP*) and FM572 (*cdc14-1 RAD52-YFP mre11Δ*) were arrested at 37°C for 3 hours and then released at 25°C. Rad52 foci were counted in dumbbells at the telophase block (0′) and in foursomes 2 and 4 hours after the release (mean ± SEM, n = 3). (B) Intensity quantification of Rad52 foci in *MRE11* and *mre11Δ* strains 2 hours after the release (see methods for details).

In the canonical model for DSB recognition and repair by HR, RPA and Rad52 are downstream players to the MRX complex (Mre11-Rad50-Xrs2) [49]. This complex is supposed to recognize each DSB end, bring them together and help in the first stages of end processing to allow template searching for HR. The expected “one-ended” nature of the DSB, a consequence of the anaphase bridge severing (i.e., the two ends cannot be brought together), prompted us to further study this important component in the DSB signalling and repair. We made use of Mre11-YFP as a reporter of the DSB-specific MRX complex. Unlike Rad52 and Rfa1, Mre11 foci have been observed in all cell cycle stages (including G1) and for all types of “two-ended” DSBs generated [46], [49]. We were not able to observe Mre11 foci for *cdc14-1* throughout the release (less than 1% of cells at any one time point in 10 minutes intervals, data not shown). Nevertheless, Mre11 was fully functional in *cdc14-1* cells growing at the permissive temperature since it forms foci when DSBs were chemically generated (Figure S9). We further ruled out any role of the MRX complex in the observed Rad52-dependent response after the release by looking at Rad52 foci in a *cdc14-1 mre11Δ* double mutant. Indeed, we saw Rad52 foci at a number and intensity comparable to that of a *cdc14-1 MRE11* strain in foursomes taken 2 and 4 hours after the telophase release (Figure 7A and 7B). Because *mre11Δ* gave some background of Rad52 foci at the *cdc14-1* arrest (Figure 7A, time 0′), we filmed cells during the release and observed that about 75% of cells with Rad52 foci at the arrest never entered a new cell cycle (data not shown). Therefore, almost all foci measured in the foursomes likely came from cells without foci at the previous arrest. We thus concluded that Rad52 foci in *cdc14-1* foursomes were independent of Mre11.

### The *cdc14-1* anaphase bridge comprises few chromosome arms aside from the chromosome XII right arm

Although the cXIIr is a hotspot for missegregation in *cdc14* mutants, it seems not to be the only genomic region affected. Thus, at least one telomere of a chromosome other than XII appeared missegregated at the telophase block in previous works that made used of another thermosensitive allele (i.e., *cdc14-3*) [22], [33]. Therefore, we decided to address whether telomeres other than cXIIr were also missegregated during the *cdc14-1* release. We looked at four different telomeres located in two chromosomes (V & XIV) [51]. Chromosome V has the right telomere labelled with the *lacO*/LacI-CFP system, whereas its left telomere (V-L) is labelled with the *tetO*/TetR-YFP system. On the other hand, chromosome XIV has the right telomere (XIV-R) labelled with the *tetO*/TetR-YFP system and its left telomere labelled with the *lacO*/LacI-CFP system. It is important to note that telomere V-L was the one used in the above-mentioned *cdc14-3* studies. When we carried out the *cdc14-1* telophase block at 37°C we often failed to detect the CFP signal, so we focused on missegregation after the release (CFP signal recovered after the temperature drop). Since the *cdc15-2* release gave only few binucleated foursomes (Figure 2 and Figure S2), and in order to avoid a possible bias, we compared the *cdc14-1* release to the *cdc15-2* telophase block. To preserve the CFP signal, we arrested *cdc15-2* cells at 34°C (at least for the YFP-labelled telomeres there was no difference between 34°C and 37°C in terms of segregation, data not shown). We observed that missegregation in *cdc14-1* binucleated foursomes was low for all four telomeres and comparable to that observed at the *cdc15-2* block (Table 1). From these data, and from the pattern of chromosome integrity shown in Figure S4, we can conclude that in a *cdc14-1* release many chromosomes are expected to be fully segregated. Thus, the anaphase bridge severed after the *cdc14-1* release must be relatively enriched with cXIIr fragments.

**Table 1:** Telomeres of chromosome arms other than cXIIr barely missegregated after a *cdc14-1* release.

	% missegregation (mean ± SD, n = 3)		p values^c^
	*cdc14-1* release^a^	*cdc15-2* block^b^	
*tel V-L*	8.23±1.35	5.82±3.93	0.372
*tel V-R*	10.59±4.85	5.39±2.78	0.1824
*tel XIV-L*	4.51±1.48	3.02±1.49	0.2865
*tel XIV-R*	4.15±2.63	3.22±1.65	0.6313

a Strains FM565 (*cdc14-1 tel V-L:tetO tel V-R:lacO tetR-YFP LacI-CFP*) and FM573 (*cdc14-1 tel XIV-L:lacO tel XIV-R:tetO tetR-YFP LacI-CFP*) were arrested in telophase (37°C) for 3 h and then released to 25°C. Samples were then taken, micrographed after DAPI staining and scored for telomere missegregation (binucleated foursomes only).

b Strains FM567 (*cdc15-2 tel V-L:tetO tel V-R:lacO tetR-YFP LacI-CFP*) and FM574 (*cdc15-2 tel XIV-L:lacO tel XIV-R:tetO tetR-YFP LacI-CFP*) were arrested in telophase (34°C) for 3 h. Samples were then taken, micrographed after DAPI staining and scored for telomere missegregation (binucleated dumbbells only).

c Statistical significance of cross-comparison between *cdc14-1* release (2 h) and *cdc15-2* block. Student's T test.

### Aberrant forms of chromosome XII can be recovered from daughter cells that survived the *cdc14-1* release

**Figure 8:**
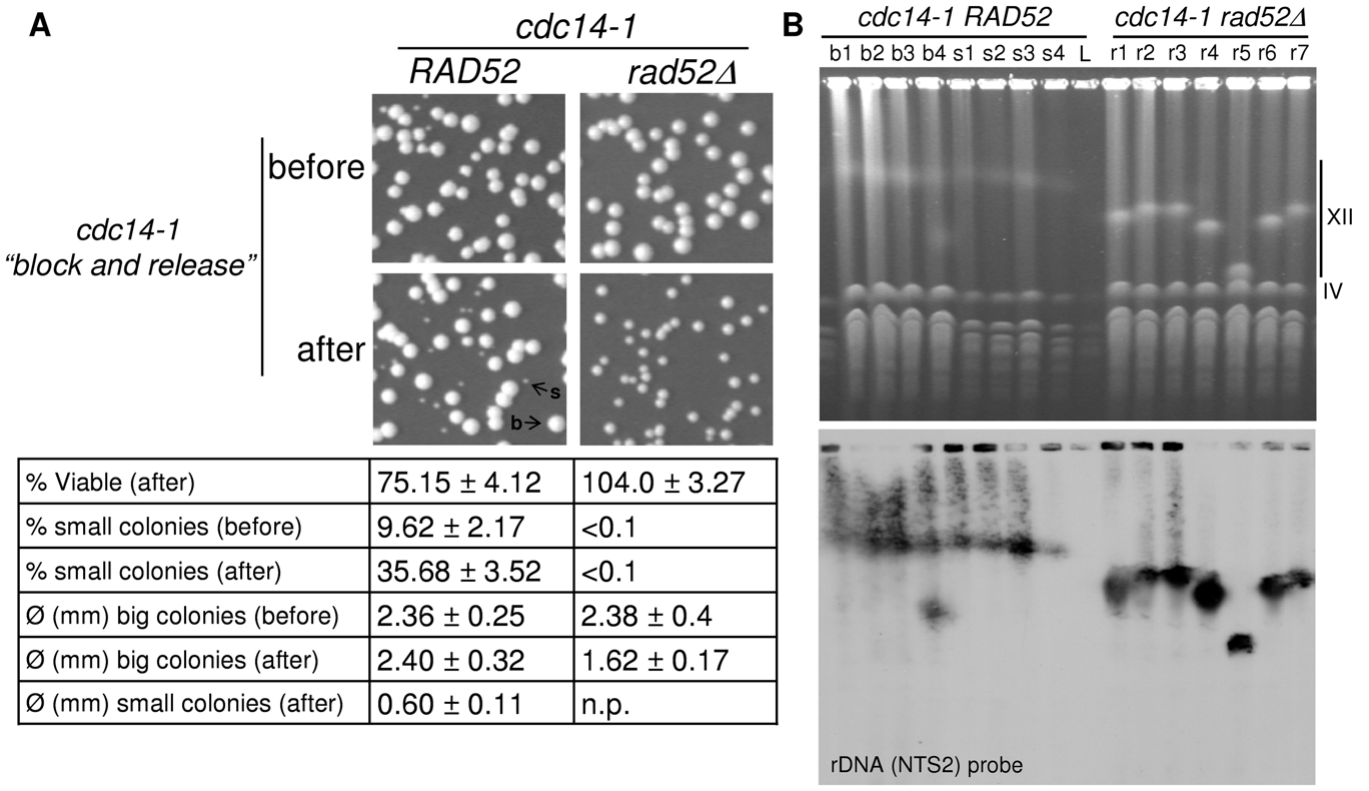
One of the two daughter cells often survives the chromosome XII missegregation event, even in the absence of Rad52. Strains FM518 (*cdc14-1 tetO:487 TetR-YFP*) and FM539 (*cdc14-1 rad52Δ tetO:487 TetR-YFP*) were arrested at 37°C for 3 hours and then released at 25°C for 2 hours. Before and after this block-and-release treatment, samples were taken and cells counted in a haemocytometer. Then, cell concentration was adjusted accordingly and serial dilution were prepared and plated on YPD. (A) Representative photos of colonies growing on YPD plates for the different strains and conditions. Examples of colonies of different sizes are indicated (“b” and “s” point to big and small colonies respectively). Underneath, a table sums up the main features of the growing colonies, including: an estimate of viable cells (actual colony number divided by cells counted before plating, mean ± SEM, n = 3), percentage of slow-growing colonies (mean ± SEM, n = 3) and colony diameter of each type (mean ± SD, n>30). Note: (i) the loss of 25% of viability after the block-and-release treatment just for *cdc14-1 RAD52* (one-way student's t test, p<0.05); (ii) the three-fold increase in very small colonies for that strain after the treatment (Student's t test, p<0.01); (iii) the clear difference of size between big and small colonies for the *cdc14-1 RAD52* strain (Student's t test, p<0.01); and (iv) the 30% decrease in colony size for *cdc14-1 rad52Δ* after the block-and-release treatment (Student's t test, p<0.01). (B) PFGE of several survivors isolated after the block-and-release treatment in both *cdc14-1 RAD52* and *cdc14-1 rad52Δ* strains. Survivors from *cdc14-1 RAD52* that formed a big colony have the prefix “b”, those where colonies were small have “s” as the prefix, and “r” was used for the *cdc14-1 rad52Δ* strain. Note how the size of chromosome XII changed in most *cdc14-1 rad52Δ* and that survivor b4 has two chromosome XIIs.

In a previous paper, we demonstrated that less than 1% of daughter cells can survive passage through multiple mitoses (>25) without Cdc14 (regulated overexpression of the cyclin-dependent kinase inhibitor Sic1 through *GAL-SIC1* was employed to overcome Cdc14 roles in the Mitotic Exit Network) [28]. In that work, we found that all survivors had dramatically shortened the rDNA locus (although other chromosome rearrangements were not obvious from the PFGE analysis). We also showed that the small survival capability depended on Rad52 (i.e., HR is needed to repair the DNA damage and survive). This prompted us to study whether our *cdc14-1* block-and-release approach, where only one cell cycle is compromised, leads to similar results. Because Rad52 also seems to play an important role after the transient Cdc14 inactivation (Figure 5, Figures S6 and S7), we also tested whether *RAD52* was essential in this system by including a double mutant *cdc14-1 rad52Δ*. Thus, we performed the block-and-release experiment for both *cdc14-1* and *cdc14-1 rad52Δ* and plated the foursomes to obtain isolated colonies after 3–5 days (Figure 8A). We also plated cells right before the block-and-release experiment, while growing asynchronously at 25°C. At the time of plating, we counted cells with a haemocytometer to determine overall viability. Surprisingly, we did not see a great loss of viability after the transient Cdc14 inactivation (Figure 8A and table underneath). As for the single *cdc14-1* mutant, this loss was around 25% at the most, whereas double mutant *cdc14-1 rad52Δ* showed no drop in viability at all. This demonstrates that at least one of the daughter cells of the foursome often survives and gives raise to a colony. Taking into account that 50% of foursomes missegregated the cXIIr (equivalent values of missegregation were seen during a *cdc14-1 rad52Δ* release: 48%), the observed percentage of viable cells might indicate that 50% and 100% of the daughter cells that carry an intact cXII (i.e., DC2) must survive in *cdc14-1* and *cdc14-1 rad52Δ* respectively. Other results we showed above already pointed towards this possibility. For instance, DC2 was often able to pass the G2/M block after a while (Figure 4B). Besides, Rad52 foci seem to eventually disappear in that cell. All these data indicate that DC2 might sometimes repair the damage and carry on dividing until it forms a colony. Importantly, we did notice that around one third of those colonies grew much more slowly in *cdc14-1* (Figure 8A, “s” colonies). These slow-growing colonies were also observed when *cdc14-1* cells where plated while normally growing at the permissive temperature. However, there was a three-fold increase in their number when plated after the transient Cdc14 inactivation (Figure 8A and table underneath). Strikingly, deletion of *RAD52* prevented these very slow-growing colonies from appearing, although most colonies grew ∼30% more slowly after the *cdc14-1* release (Figure 8A and table underneath).

The different effects of both the block-and-release experiment and the presence of Rad52 on the colony size of survivors prompted us to analyse the state of chromosome XII in the different outcomes (Figure 8B). Thus, we grew several colonies at the permissive temperature and carried out PFGE. We found that chromosome XII was shorter in the *cdc14-1 rad52Δ* strain we used (Figure 8B) than in its parental *cdc14-1 RAD52* strain. Interestingly, chromosome XII was highly unstable in the *cdc14-1 rad52Δ* survivors, whereas it remained more constant in *cdc14-1 RAD52*, even in the slow-growing survivors (Figure 8B). One of the *cdc14-1 RAD52* survivors (#b4) could have duplicated chromosome XII as suggested by the presence of two rDNA-containing bands.

From this set of experiments we conclude that many foursomes where cXIIr missegregation occurred can still carry on dividing for many generations (DC2 likely seeds these survivors). In addition to this, chromosome XII rearrangements and a reduced fitness are frequent outcomes of transient inactivation of Cdc14 for one cell cycle.

## Discussion

### The *cdc14-1* release experiment as a model to study severing of anaphase bridges comprising unresolved sister chromatids

The major manifestation of entering anaphase without completing sister chromatid resolution is the appearance of anaphase bridges. Herein, we have introduced a new model to study the short-term consequences of these bridges based on the primary phenotype observed for the *cdc14-1* mutant of *Saccharomyces cerevisiae* [23], [24], [28]. From a technical point of view this model presents several key advantages that facilitate cell biology studies on anaphase bridges: (i) non-resolution specificity for few genomic regions (e.g., cXIIr, see below); (ii) cell mixtures of segregated and missegregated cXIIr in the same population and experiment [28] (Figure 2); (iii) synchrony of the cells exiting mitosis (Figure 2); (iv) capability to monitor and cross-compare both daughter cells as they remain together after a *cdc14-1* release (Figure 2 and Figure 3); and (v) availability of a proper parallel control that mostly behaves like *cdc14-1* but does segregate the cXIIr (i.e., *cdc15-2* conditional allele) [22] (Figure 2, Figures S1 and S4).

### The *cdc14-1* anaphase bridge and its fate in comparison to what is observed in separase, condensin, and *top2* mutants

**Figure 9:**
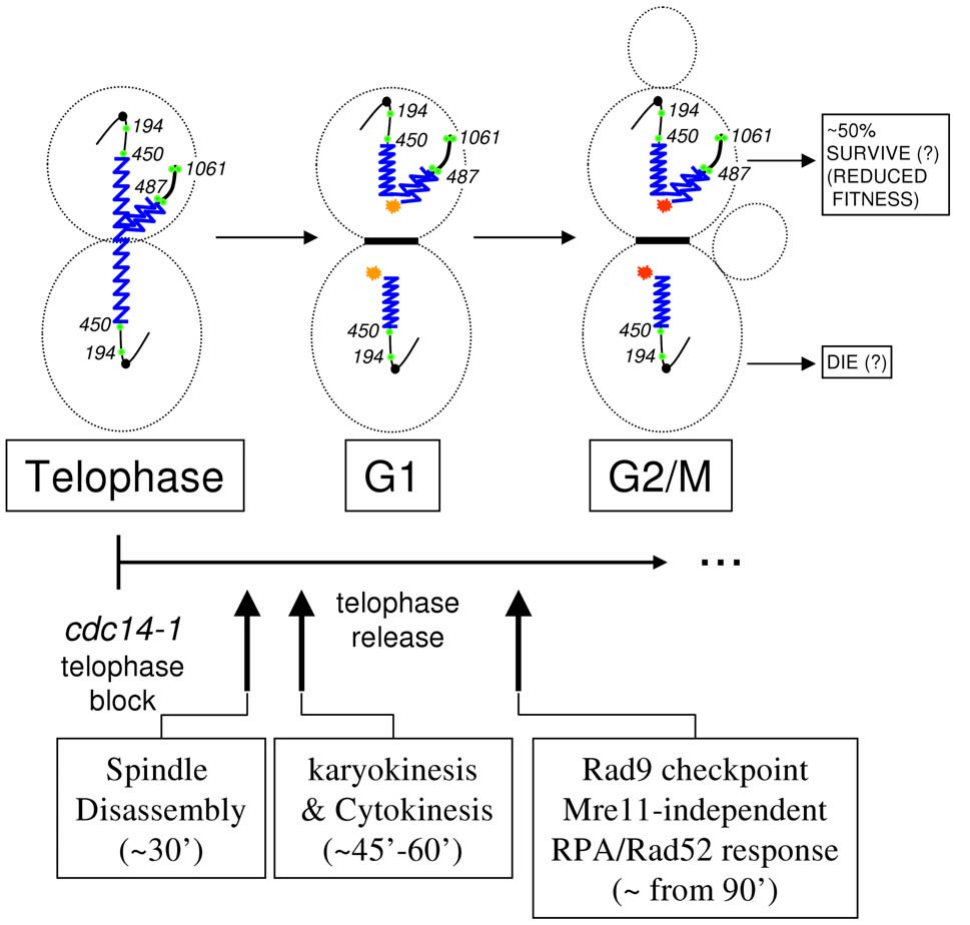
Model of how the cell cycle progresses after the presence of the cXIIr bridge. Location of *tetOs* is shown as green dots; rDNA is depicted as a serrated blue line; and thicker lines indicate non-resolved sister chromatids. Stars indicate the expected “one-ended” double strand break (orange star means that the break does not elicit a strong DNA damage response; whereas red star means it does). Main cell cycle events described in this article are indicated in a time line underneath. The likely long-term fate of each daughter cell (as deduced from Figure 8) is also indicated.

Because Cdc14 controls condensin and Top2 in anaphase and directs their activities to the rDNA [22], [27], [28], [30], [31], the overall expectation of our system is that the *cdc14-1* anaphase bridge is like those of condensin and *top2* mutants, however mainly restricted to a single chromosome arm (i.e., cXIIr). Therefore, it is interesting to compare our results to those previously reported for condensin and *top2* mutants. We also include here mutants for cohesin removal due to their similarities. All these mutants form anaphase bridges comprised of trailing and distally unresolved sister chromatids as we depict in Figure 1 and Figure 9 (just for chromosome XII in those figures [i.e., *cdc14-1*], more chromosome arms are like cXIIr for these other mutants). This pattern of non-resolution likely arises from the spindle forces being able to slide cohesin and catenations away from bipolarly attached centromeres. Importantly, these mutants differ in the extent of non-resolution along the chromosome arms and the number of affected chromosomes, cohesin-removal mutants having the strongest phenotype and *cdc14-1* the mildest [14], [19], [20], [23]. Accordingly, a common outcome in cells where cohesin or catenation removal have been impaired is the appearance of an anaphase where the nuclear mass cannot be split in two. For instance, condensin or *top2* conditional mutants show rod-like nuclei in anaphase [15], [16]. The same outcome is seen in mutants where cohesin cleavage is inhibited in anaphase (e.g., separase mutants or non-cleavable forms of cohesin) [14]. Importantly, this unresolved nucleus does not abort cytokinesis, which eventually takes place leading to a “cut” phenotype in all cases. This phenotype is characterized by aneuploid daughter cells carrying broken chromosomes [14], [16], [32]. Another common feature of those daughter cells is that many are unable to resume the cell cycle, likely because of the massive chromosome breakage observed. The results we present in this work indicate that the anaphase bridge in *cdc14-1* and its fate is somewhat different. First, the *cdc14-1* block does not lead to a rod-like nucleus in anaphase, rather it is able to split the two DNA masses, which end up in each daughter cell [23]–[25], [34]. Likely, this is the consequence of most chromosome arms being able to segregate at the block. It is important to point out that we have assessed four telomeres of two other chromosomes (V and XIV) and found little missegregation in *cdc14-1* foursomes relative to a *cdc15-2* block (Table 1). Moreover, the drop of band intensity in the PFGE was only seen for chromosome XII in the *cdc14-1* release (Figure 2E and Figure S4). Taking into account that, even in *top2* and condensin mutants, small and medium-sized chromosomes segregate despite the rod-like nuclear phenotype [19], [21], we believe that the anaphase bridge in *cdc14-1* mutants must comprise few chromosome arms; and that those severed by cytokinesis after the *cdc14-1* release are fewer than for the other mutants. This in turn would explain why both daughter cells reach G2/M (Figure 2 and Figure S2). If more than four chromosome arms were severed, we would expect a G1 delay [45], which we did not observe.

On the other hand, in all mutants but *cdc14-1*, cytokinesis is followed by cell separation. This makes difficult to follow up and cross-compare both daughter cells, a key advantage we show for *cdc14-1*. It is unclear why *cdc14-1* daughter cells are unable to separate from each other. However, this phenotype can be also seen when overcoming the *cdc14-1* block by overexpressing Sic1 [28], [52]; and it was then shown that cytokinesis was completed [52]. Our data also suggest that cytokinesis is completed after *cdc14-1* re-activation (Figure 3). Thus, a possible role of Cdc14 in cell septation could be responsible for this phenotype (Figure 2). A role that could actually be extended to the mitotic exit network as *cdc15-2* also has a partial defect in cell separation.

Finally, the anaphase bridges formed in cohesin and some *top2* mutants have been employed to define a checkpoint that delays cytokinesis (i.e., NoCut checkpoint) [37]. The actual length of such delay has been difficult to measure. In our case, the *cdc14-1* anaphase bridge gave a short delay of about 20 minutes when comparing karyokinesis relative to the maintenance of the cXIIr bridge after the release (Figure 3B), and likely accounts for the NoCut checkpoint. Nevertheless, this delay was difficult to see by other means. For instance, it was observed neither relative to *cdc15-2* (at least in the S288C background) nor as a biphasic drop of dumbbell cells in *cdc14-1* (i.e., cells able to segregate the cXIIr versus those with the cXIIr bridge) (Figure 2A and Figure S2). Besides, the dynamics of the spindle disassembly after the release were quick for dumbbell cells (within the first hour, Figure S3A, left panel). It may be possible that a greater number of chromosomes in the anaphase bridge obtained by other mutants may trigger a stronger checkpoint signal.

### The *cdc14-1* anaphase bridge and its fate in comparison to bridges of a different physical nature

As stated above, the *cdc14-1* anaphase bridge is supposed to be similar to condensin and *top2* mutants, yet restricted to cXIIr. Notably, there are other situations where we can predict anaphase bridges of a different nature. For instance, anaphase bridges formed by partly replicated chromatids which nevertheless enter anaphase. For instance, *sic1* and *smc5/6* mutants behave this way [53], [54]. As with the difference between *top2* and *cdc14-1*, *sic1* and *smc5/6* also differ in the actual number of chromosomes in the bridge, cXIIr being enriched in mutants for the Smc5/6 complex [44], [53], [54]. Despite the cytological similarities of the anaphase bridges between *top2* and *sic1*, and between *cdc14-1* and *smc5/6*, the bridge appeared broken in anaphase before completing cytokinesis in *sic1* and *smc5/6* mutants and a DDR can be also observed in that cell cycle stage [53], [54]. These findings highlight a key difference between the anaphase bridges formed by tangled sister chromatids and those where replication is incomplete: breakage before cytokinesis occurs in the latter, perhaps due to more fragile DNA in the unreplicated material. Finally, it is interesting to point out that other *cdc14* mutants might enter anaphase with unreplicated DNA as well [33]. However, the behaviour of our *cdc14-1* bridge is much closer to *top2* and condensin than to what is observed in *sic1* and *smc5/6*. This is, the chromosome can enter a PFGE in the *cdc14-1* block (Figure 2E) and no DDR is observed at the block (Figure 5A and Figure 6A).

Another distinct anaphase bridge is that accomplished by the use of conditional dicentric chromosomes [55]–[57]. Like our *cdc14-1* model, this approach has multiple technical advantages such as: (i) an anaphase bridge formed by a single chromosome; and (ii) cells with and without the bridge in the same population and experiment (∼50% chance of having two centromeres within a single sister chromatid attached to opposing SPBs). Nevertheless, the physical nature of the bridge is rather different. In the dicentric model, the bridge is formed by the sister chromatids being in an anti-parallel conformation. Moreover, sisters are supposed to be completely resolved from each other. In *cdc14-1*, the bridge is often formed by just one sister, the other one being out of the cytokinetic plane (Figure 1 and Figure 9) [23]. Thus, the expected DSBs and the broken genetic material in daughter cells are different when a dicentric chromosome is used. In relation to this system, it is interesting to note that the conditional dicentric chromosome triggers a Rad9-dependent mid-anaphase checkpoint (characterized by short spindles) that we did not see (data not shown) [24], [56]. Regardless, this and other dicentric models, like *top2*/condensin/*cdc14-1* mutants, do not seem to break the anaphase bridge until cytokinesis takes place [57], [58].

### On the DNA damage generated after severing the *cdc14-1* chromosome XII right arm anaphase bridge

A key conclusion of this work is that at least one DSB near or within the rDNA is produced after the release from the *cdc14-1* block (Figure 2, Figure 3, Figure 4). Unlike DSBs generated by endonucleases, radiation or any other means within a single nucleus [49], DSBs generated during anaphase bridge severing cause the ends of the broken DNA molecule(s) to migrate to opposing compartments which cannot be brought together anymore (i.e., nuclei of daughter cells). In the first scenario, the two ends of the DSB can be physically tied again and repaired by either non-homologous end joining (NHEJ) or HR. However, in the second case, the severing of the DNA molecule during nuclear division leads to a DSB where only one end can be found in each daughter nucleus (i.e., a “one-ended” DSB). Importantly, the state of each daughter cell is actually different with regard to chromosome XII dose (see Figure 1 and Figure 9 for schemes). While one cell (i.e., “DC2”) would have an entire chromosome XII plus the broken distal region of the right arm of the same chromosome (from the DSB to the telomere), the other one (i.e., “DC1”) would retain a fragment of a single chromosome XII (from the left telomere to the DSB, including its centromere). It is difficult to envisage how each DSB end might be repaired. For instance, break-induced replication, *de novo* telomere addition, chromosome translocation and/or elimination of the broken sisters might well be possible. Confounding matters, if the DSB takes place within the rDNA, which may happen often according to our data, cells can find a template for HR in another copy of the array. This latter situation can lead to an uncertain outcome (e.g., extrachromosomal circles?). Whichever way daughter cells face the problem, our results provide several interesting observations: i) the DSB(s) does not trigger a strong DDR in the new G1 (Figure 2 and Figure 6); ii) the MRX complex (i.e., Mre11) has no role in DSB(s) processing (Figure 7 and Figure S9); iii) the Rad9 checkpoint protein, the RPA complex and Rad52 are part of the mechanism to deal with these DSBs as soon as both daughter cells reach S-phase (Figure 4, Figure 5, Figure 6, Figure S6, and Figure S7); iv) the activity of these key proteins is long lasting and cumulative, especially in the daughter cell that only carries a fragmented cXIIr copy (i.e., DC1) (Figure 4, Figure 5, Figure 6); and v) DC2 often survives and might get rid of the broken distal fragment of cXIIr in order to do so (without using it as a template for HR, see below and Figure 8).

In relation to the absence of both a G1 arrest and Rfa1 foci in the new G1, our results indicate that the DSB generated after *cdc14-1* release is similar to that generated by endonucleases (i.e., a “clean” DSB) as opposed to those generated by ionizing radiation (i.e., “ragged” DSBs) [45], [46], [49]. Also, it indicates that the number of DSBs should be below four or five (i.e., few chromosome arms are part of the anaphase bridge) [45]. Besides this, it is interesting that the processing of these DSBs is independent of Mre11 (Figure 7). Perhaps the MRX complex is not needed because the one-ended nature of each DSB means that there is no need to join both broken ends. Perhaps other molecular players are required in this context. In any case, others have previously reported that cells deficient in Mre11 and other MRX components can still generate a strong DDR and repair DSB through HR [59]. An alternative explanation for the Rfa1/Rad52 foci would be related to a sort of *de novo* damage generated as a consequence of DNA replication through an unrepaired or faultily-repaired chromosome XII. Future works are also needed in this direction.

As for the long-term consequences of this type of one-ended DSB, it is interesting that we observed that the DC2 cell can eventually recover in many cases (Figure 4 and Figure 8). Most surprising was the fact that viability got better when *RAD52* was deleted. We interpret this as indicating that checkpoint adaptation followed by loss of the acentric fragment might be the main pathway that allows DC2 to progress. Accordingly, surviving *cdc14-1 rad52Δ* foursomes grew ∼30% more slowly (they actually took around one extra day to be visible on plates) relative to the same cells plated before the block-and-release experiment (Figure 8A). Indeed, the presence of Rad52 might compromise the chances of *cdc14-1* DC2 cells surviving with a good fitness (Figure 8A). Contrary to expectations, though, PFGE of survivors showed that chromosome XII was more unstable in *cdc14-1 rad52Δ.* Also, the slow-growing colonies of *cdc14-1 RAD52* did not show visible abnormal chromosome patterns. Despite our having checked only a few surviving colonies, it is interesting that none show evidence of chromosome XII rearrangements that involve translocations, although one might have duplicated the chromosome. Thus, we concluded that the DC2 cell very often survives and that it might repair the broken cXIIr in two ways; one which is dependent on Rad52 (e.g., through break induced replication, a likely event at least in the survivor with two chromosome XIIs), and a second Rad52-independent manner that somehow makes more likely changes in chromosome XII size.

As far as we know, this is the first time that an analysis of the DNA damage generated by cytokinetic severing of a single chromosome is conducted in yeast. A recent paper has just described the DDR after cytokinesis severs lagging chromosomes in human cells [60]. Many conclusions from that paper agree with those we observed in our work, although the system is clearly distinct (i.e., more than one chromosome is affected, both sister chromatids are severed, etc). In these human cells, DSBs arise after cytokinesis and are often repaired by NHEJ in G1, leading to aberrant chromosomes. The difference in the mechanism of repair in our yeast system is nevertheless expected, since yeast basically rely on HR acting through S and G2 rather than NHEJ in G1 [42]. Another key difference between both systems is the ploidy of the dividing cells. Human cells are diploids and may repair broken sisters using homologous chromosomes as templates. Our yeast strains were all haploids. It would be interesting to study whether *cdc14-1* diploids also missegregate cXIIr and whether the DDR is different from what we describe here for haploids. Future work will be carried out to this aim.

### Conclusion

In this study we have assessed the fate of cells that have an anaphase bridge formed by the right arm of chromosome XII (Figure 9 for a model and summary). We show how cells can go through a new G1, although they sever the bridge as they complete cytokinesis, and reach G2/M where they get arrested in a Rad9-dependent manner. We also show that the expected DNA damage response comprised RPA and Rad52, but is independent of Mre11. All these data shed light on how one-ended DSBs generated by a “cut” phenotype may be processed in eukaryotic cells. This work provides the first systematic study of the cell responses to a previous failure in sister chromatid resolution.

## Materials and Methods

### Yeast strains, growth, and experimental conditions

All yeast strains used in this work are listed in Table 2. Strains with the *tetOs* along chromosome XII right arm and those with tags for chromosome XIV telomeres were S288C background. Those with Rad52-YFP, Rfa1-YFP, Mre11-YFP, GFP-Tub1 and Tub4-CFP tags, and those with tags for chromosome V telomeres were W303. C-terminal tagging with GFP variants, gene deletions and allele replacements were performed using PCR methods [61], [62]. All strains were grown overnight at 25°C in YPD media. For telophase block-and-release experiments, asynchronous cultures were first adjusted to OD_660_/ml = 0.2, incubated at 37°C for 3 h in air orbital incubators and then shifted back to 25°C. To arrest cells in G1 in the cell cycle that follows the telophase release, cells were treated with alpha-factor (50 ng/ml) for 2 hours after the 25°C shift (all tested strains were *bar1Δ*). Flow cytometry analysis was carried out as described [54] in a BD FACScalibur machine, adjusting the peaks for 1 N and 2 N with an asynchronous culture at 25°C before reading the samples. PFGE to see all yeast chromosomes was performed using a CHEF DR-III system (Bio-Rad) in a 0.8% agarose gel in 0.5× TBE buffer and run at 12°C for 20 h at 6 V/cm with an initial switching time of 80 seconds, a final of 150 seconds, and an angle of 120°. PFGE to assess the size of chromosome XII was performed at 3 V/cm for 68 h with 300 and 900 seconds of initial and final switching time respectively. Ethidium bromide was used to visualize the chromosome bands in the gel. Band quantifications were performed with ImageJ software (NIH). Chromosome XII band(s) was identified by Southern blot using a Digoxigenin-labelled probe (Roche) against the NTS2 region within the rDNA.

**Table 2:** Strains used in this work.

Strain name	Relevant genotype	Origin
AS499	(S288C) *bar1Δ*	A. Strunnikov
GA2199	(W303) *BAR1 Telomere V-L:TetO TetR-YFP Telomere V-R:lacO CFP-lacI*	S. Gasser
GA2468	(S288C) *BAR1 Telomere XIV-L:lacO Telomere XIV-R:tetO CFP-lacI TetR-YFP*	S. Gasser
W3749-14c	(W303) *bar1Δ RAD5 RAD52-YFP*	D. Rothstein
W3775-12c	(W303) *bar1Δ RAD5 RFA1-YFP*	D. Rothstein
W3483-10a	(W303) *bar1Δ RAD5 MRE11-YFP*	D. Rothstein
ML118-1D	(W303) *BAR1 RAD5 tetOx224:rDNA TetR-mRFP RAD52-YFP*	M. Lisby
DOM0114	(W303) *bar1Δ cdc15-2*	D. Morgan
MGY146a	(W303) *bar1Δ cdc14-1*	C. Nombela
FM304 (CCG1605)	AS499 *tetOx224:chrmXII(194 Kb) TetR-YFP cdc14-1 NET1-CFP*	L. Aragon
FM307 (CCG1607)	AS499 *tetOx224:chrmXII(450 Kb) TetR-YFP cdc14-1 NET1-CFP*	L. Aragon
FM518 (CCG1679)	AS499 *tetOx224:chrmXII(487 Kb) TetR-YFP cdc14-1*	L. Aragon
FM322 (CCG1609)	AS499 *tetOx224:chrmXII(1061 Kb) TetR-YFP cdc14-1 NET1-CFP*	L. Aragon
FM593	AS499 *tetOx224:chrmXII(194 Kb) TetR-YFP cdc15-2*	This work
FM582	AS499 *tetOx224:chrmXII(450 Kb) TetR-YFP cdc15-2*	This work
FM584	AS499 *tetOx224:chrmXII(487 Kb) TetR-YFP cdc15-2*	This work
FM588	AS499 *tetOx224:chrmXII(1061 Kb) TetR-YFP cdc15-2*	This work
FM459	MGY146a *GFP-TUB1 cdc14-1*	This work
FM576	MGY146a *GFP-TUB1 cdc14-1 rad9Δ*	This work
FM458	MGY146a *TUB4-CFP cdc14-1*	This work
FM565	GA2199 *Tel V-L:TetO TetR-YFP Tel V-R:lacO CFP-lacI cdc14-1*	This work
FM573	GA2468 *Tel XIV-L:lacO Tel XIV-R:tetO CFP-lacI TetR-YFP cdc14-1*	This work
FM567	GA2199 *Tel V-L:TetO TetR-YFP Tel V-R:lacO CFP-lacI cdc15-2*	This work
FM574	GA2468 *Tel XIV-L:lacO Tel XIV-R:tetO CFP-lacI TetR-YFP cdc15-2*	This work
FM515	W3749-14c *RAD52-YFP cdc14-1*	This work
FM531	W3749-14c *RAD52-YFP cdc15-2*	This work
FM547	W3749-14c *RAD52-YFP cdc14-1 fob1Δ*	This work
FM883	W3749-14c *RAD52-YFP cdc14-1 rad9Δ*	This work
FM460	W3749-14c *RAD52-YFP cdc14-1 NOP1-DsRed*	This work
FM551	ML118-1D *tetOx224:rDNA TetR-mRFP RAD52-YFP cdc14-1*	This work
FM753	AS499 *tetOx224:chrmXII(487 Kb) TetR- YFP cdc14-1 RAD52-RedStar2*	This work
FM513	W3775-12c *RFA1-YFP cdc14-1*	This work
FM514	W3483-10a *MRE11-YFP cdc14-1*	This work
FM572	W3749-14c *RAD52-YFP cdc14-1 mre11Δ*	This work
FM539	AS499 *tetOx224:chrmXII(487 Kb) TetR-YFP cdc14-1 rad52Δ*	This work

### Fluorescence microscopy

Fluorescent proteins and chromosome tags were analysed by wide-field fluorescence microscopy. Series of z-focal plane images (10–20 planes, 0.15–0.3 µm depth) were collected on a Leica DMI6000, using a 63×/1.30 immersion objective and an ultrasensitive DFC 350 digital camera, and processed with the AF6000 software (Leica). Scale bars in micrographs depict 5 µm. For nuclear morphology studies, DNA was stained using DAPI at 4 µg/ml final concentration after short cell treatment with 1% Triton X-100. Time-lapse movies were filmed without Triton/DAPI treatment on minimal medium agarose patches. Imaging was done at room temperature. Nucleoplasm pictures using nuclear-tagged TetR-YFP was also done without Triton/DAPI treatment. Rad52 foci recognition was performed either manually or using the CellProfiler software [63]. For the latter, whole images were normalized following the procedure: most intense focus in the first photo taken was set to 1, least intense pixel of the background was set to 0. A lower threshold of 0.1 was set for foci recognition.

### Cytokinesis assays

Cytokinesis was monitored as previously described [39] with minor modifications. Briefly, aliquots of cells were fixed directly in the growth media by the addition of formaldehyde to 5% final concentration. After incubation at 25°C for 1 h with gentle rocking, fixed cells were washed twice with PBS and then once with 1 M sorbitol in 50 mM KPO_4_, pH 7.5. Cells were incubated with 0.2 mg/ml zymolyase 20T (Zymo Research) in the above sorbitol buffer containing 2 mM DTT for 20 minutes at 37°C. After zymolyase treatment, cell numbers were counted on a haemocytometer.

Special chemical treatments in these assays (i.e., nocodazole, alpha-factor and latrunculin A) were performed as follows: The arrest in G2/M was carried with 15 µg/ml of nocodazole at 25°C for 2.5 hours. Latrunculin A (100 µM) was added right at the G2/M release and alpha-factor (50 ng/ml) was added right at the telophase release. Release from the G2/M arrest was accomplished by washing away the nocodazole. Incubation for 1.5 hours at 37°C was used to block cells in telophase after a G2/M release. For the telophase release, cultures were shifted back to 25°C. Samples for this cytokinesis assay were taken at the telophase block and two hours after the telophase release. We used a *cdc14-1* strain of the W303 background in these assays because it gave better synchrony, especially during the double block-and-release experiments (first at G2/M and then at telophase).

### Statistics

Error bars in graphs represent the standard error of the mean (SEM) unless stated otherwise. The number of experiments is indicated in the corresponding figure legend or table. Statistic inference for cross-comparison of categorical variables distributions were performed by the Fisher's exact test when a 2×2 contingency table could be built (e.g., segregation vs. missegregation). For other categorical variables with more than two possible outcomes (e.g., number of Rad52 foci), the Pearson's chi-square test was employed. Individual comparisons between means of independent experiments were performed by the Student's T test. All tests were two-tailed.

## Supporting Information

Figure S1 Cells faithfully segregate chromosome XII in a *cdc15-2* telophase block. Strains FM304 (*cdc14-1 tetO:194 TetR-YFP*), FM307 (*cdc14-1 tetO:450 TetR-YFP*), FM518 (*cdc14-1 tetO:487 TetR-YFP*), FM322 (*cdc14-1 tetO:1061 TetR-YFP*), FM593 (*cdc15-2 tetO:194 TetR-YFP*), FM582 (*cdc15-2 tetO:450 TetR-YFP*), FM584 (*cdc15-2 tetO:487 TetR-YFP*) and FM588 (*cdc15-2 tetO:1061 TetR-YFP*) were arrested at 37°C for 3 hours and resolution and segregation status of *tetOs* (mean ± SEM, n = 3) were scored for dumbbell binucleated cells (>200 cells each).
(TIF)

Figure S2 Cells do not enter anaphase after a *cdc14-1* release in the W303 background. Strains DOM0114 (*cdc15-2*) and MGY146a (*cdc14-1*) were arrested in telophase by incubation at 37°C for 3 hours (time = 0′) and then released from the arrest by dropping the temperature to 25°C. Samples were taken every 15–30 minutes for 4 hours, stained with DAPI and analysed by microscopy for budding pattern (upper panels) and nuclear morphology (lower panels). For the nuclear morphology analysis only daughter cells that have rebudded are included and each daughter is counted individually for simplicity. Note how *cdc15-2* gave an oscillatory behaviour indicative of cells cycling; whereas *cdc14-1* got stuck as foursomes with just two nuclear masses (one mass per daughter cell).
(TIF)

Figure S3 A *cdc14-1* release leads to daughter cells stuck with metaphase spindles. (A) Strain FM459 (*cdc14-1 TUB1-GFP*) was treated as in Figure S2 and cells were scored for spindle morphology in either unbudded dumbbells (left panel) or rebudded daughter cells (right panel) (mean ± SEM, n = 3). Each rebudded daughter was counted as an individual new cell. (B) Strain FM458 (*cdc14-1 TUB4-CFP*) was arrested in telophase by incubation at 37°C for 3 hours (time = 0′) and then released from the arrest by dropping the temperature to 25°C. Samples taken 2 hours after the shift (120′) were stained with DAPI and analysed by microscopy. Note: Around 80% of nuclear masses have two CFP foci. Bar, 5 µm.
(TIF)

Figure S4 Chromosome band quantification of *cdc15-2* and *cdc14-1* telophase releases. The pulsed-field gel depicted in the upper panels of Figure 3B and two more independent experiments were scanned to quantify each chromosome band and normalized to that at the telophase block (mean ± SEM, n = 3). In the graphs we show the results for the two largest chromosomes (XII and IV) and for other bands containing medium size chromosomes. Note how all chromosomes entered a successful replication round (i.e., bands faded away and came back later) for both mutants; whereas chromosome XII dropped shortly after the *cdc14-1* release (minute 60) and never came back in full. Also note how this drop was observed when replication was prevented by releasing into α-factor (G1 column).
(TIF)

Figure S5 The nucleoplasm bridge of soluble TetR-YFP as seen in the *cdc15-2* and *cdc14-1* telophase blocks. (A) Strains FM584 (*cdc15-2 tetO:487 TetR-YFP*) and FM518 (*cdc14-1 tetO:487 TetR-YFP*) were arrested at 37°C for 3 h and micrographed. (B) Strain FM304 (*cdc14-1 tetO:194 TetR-YFP NET1-CFP*) was arrested as in A. Each photo represents different Z-stacks in 0.3 µm intervals. Hollow triangles point to the nucleoplasm bridge. Filled triangles point to the bulge in the bridge observed at the *cdc14-1* block. Bar, 5 µm. Note how the nucleoplasm bridge is seen in all cells at both telophase blocks, the bulge is seen only in *cdc14-1*, and that the bulge contains the bulk of the rDNA (Net1-CFP).
(TIF)

Figure S6 Worsening of chromosome XII segregation through deletion of *FOB1* increases the number of Rad52 repair factories. Strains FM515 (*cdc14-1 RAD52-YFP*) and FM547 (*cdc14-1 fob1Δ RAD52-YFP*) were first arrested in the *cdc14-1* block and then released into a new cell cycle. After 2 hours, foursomes were scored for number of Rad52 foci (mean ± SEM, n = 3). Note how foursomes with no Rad52 foci dropped from ∼50% to ∼20% when the *fob1Δ* mutation was present (rDNA missegregation increased from ∼50% to ∼95% relative to *FOB1*).
(TIF)

Figure S7 Presence of Rad52 repair factories correlates to previous failure in rDNA segregation after a *cdc14-1* release. Strain FM753 (*cdc14-1 RAD52-RedStar2 tetO:487 tetR-YFP*) was first arrested in the *cdc14-1* block and then released into a new cell cycle. After 2 hours, Rad52 foci were scored for those foursomes that have either segregated or missegregated the *tetO* (mean ± SEM, n = 3).
(TIF)

Figure S8 Rad52 repair factories localize out of the nucleolus after a *cdc14-1* release. Strain FM460 (*cdc14-1 RAD52-YFP NOP1-DsRed*) was first arrested and micrographed in the telophase block (0′) and then two hours after the release (120′). Representative micrographs of the major cell types are shown. In the channel composite, DAPI is pseudocoloured in red and Nop1 in blue. Note how Rad52 foci in foursomes (at 120′) do not colocalize with the nucleolar marker Nop1.
(TIF)

Figure S9 Mre11 is functional under the *cdc14-1* background and concentrates in nuclear factories after chemically-generated DNA double strand breaks. Strain FM514 (*cdc14-1 MRE11-YFP*) was grown at 25°C until log phase and directly treated with either 25 µg/ml phleomycin or 0.03% v/v MMS. Then, samples were taken every 10 minutes and micrographed under the microscope. Mre11-YFP started concentrating in foci after just 20 minutes. Example micrographs taken after 2 hours of treatments are shown. White filled triangles point to Mre11-YFP foci.
(TIF)
