# Supplementary figures and images for: Nondisjunction of a Single Chromosome Leads to Breakage and Activation of DNA Damage Checkpoint in G2
Source: PLoS Genet. 2012 Feb 16;8(2):e1002509. doi: 10.1371/journal.pgen.1002509 (PMC3280967; doi:10.1371/journal.pgen.1002509)

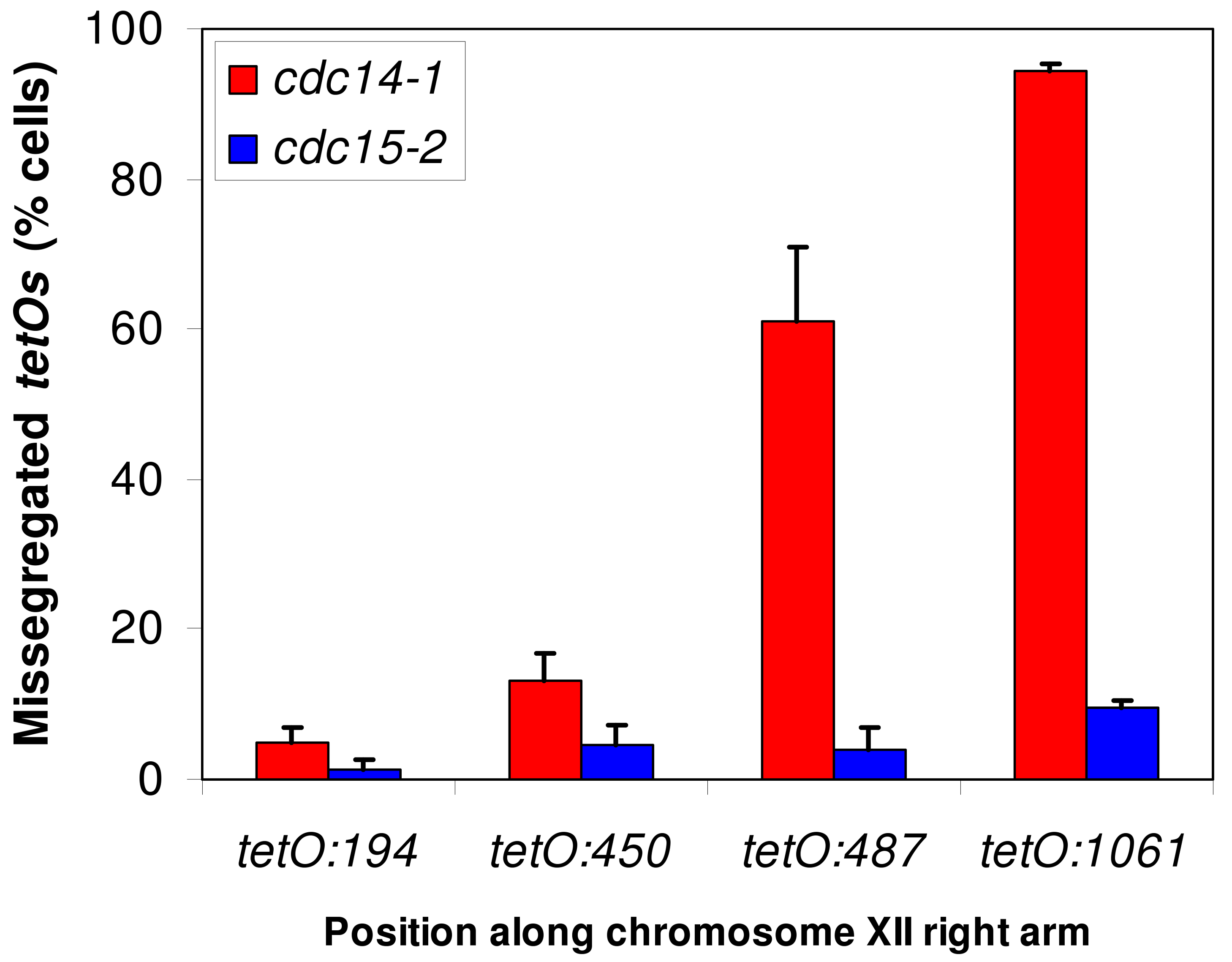

Supplement: Figure S1 — Cells faithfully segregate chromosome XII in a cdc15-2 telophase block. Strains FM304 (cdc14-1 tetO:194 TetR-YFP), FM307 (cdc14-1 tetO:450 TetR-YFP), FM518 (cdc14-1 tetO:487 TetR-YFP), FM322 (cdc14-1 tetO:1061 TetR-YFP), FM593 (cdc15-2 tetO:194 TetR-YFP), FM582 (cdc15-2 tetO:450 TetR-YFP), FM584 (cdc15-2 tetO:487 TetR-YFP) and FM588 (cdc15-2 tetO:1061 TetR-YFP) were arrested at 37°C for 3 hours and resolution and segregation status of tetOs (mean ± SEM, n = 3) were scored for dumbbell binucleated cells (>200 cells each). (TIF) [file pgen.1002509.s001.tif]

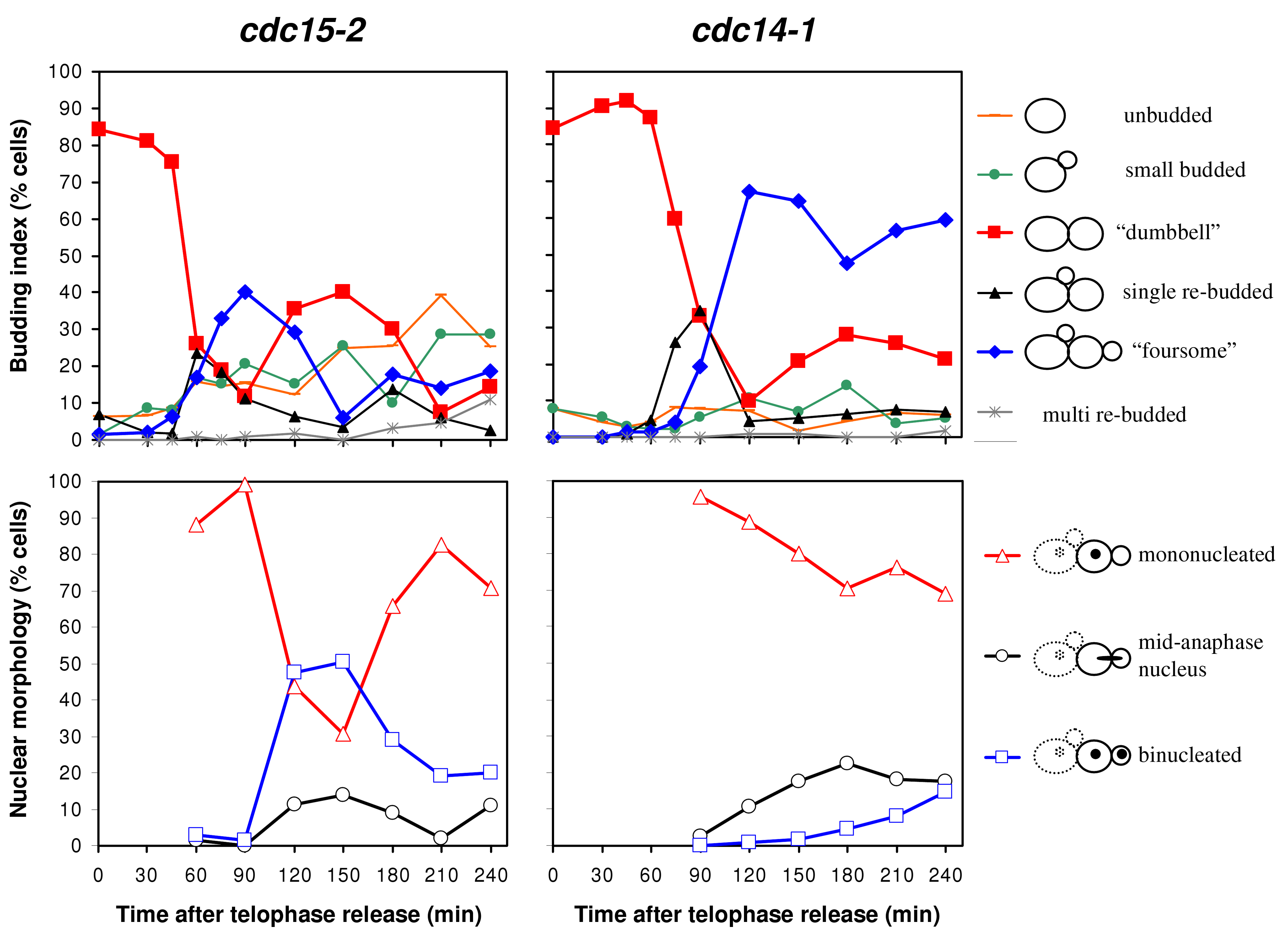

Supplement: Figure S2 — Cells do not enter anaphase after a cdc14-1 release in the W303 background. Strains DOM0114 (cdc15-2) and MGY146a (cdc14-1) were arrested in telophase by incubation at 37°C for 3 hours (time = 0′) and then released from the arrest by dropping the temperature to 25°C. Samples were taken every 15–30 minutes for 4 hours, stained with DAPI and analysed by microscopy for budding pattern (upper panels) and nuclear morphology (lower panels). For the nuclear morphology analysis only daughter cells that have rebudded are included and each daughter is counted individually for simplicity. Note how cdc15-2 gave an oscillatory behaviour indicative of cells cycling; whereas cdc14-1 got stuck as foursomes with just two nuclear masses (one mass per daughter cell). (TIF) [file pgen.1002509.s002.tif]

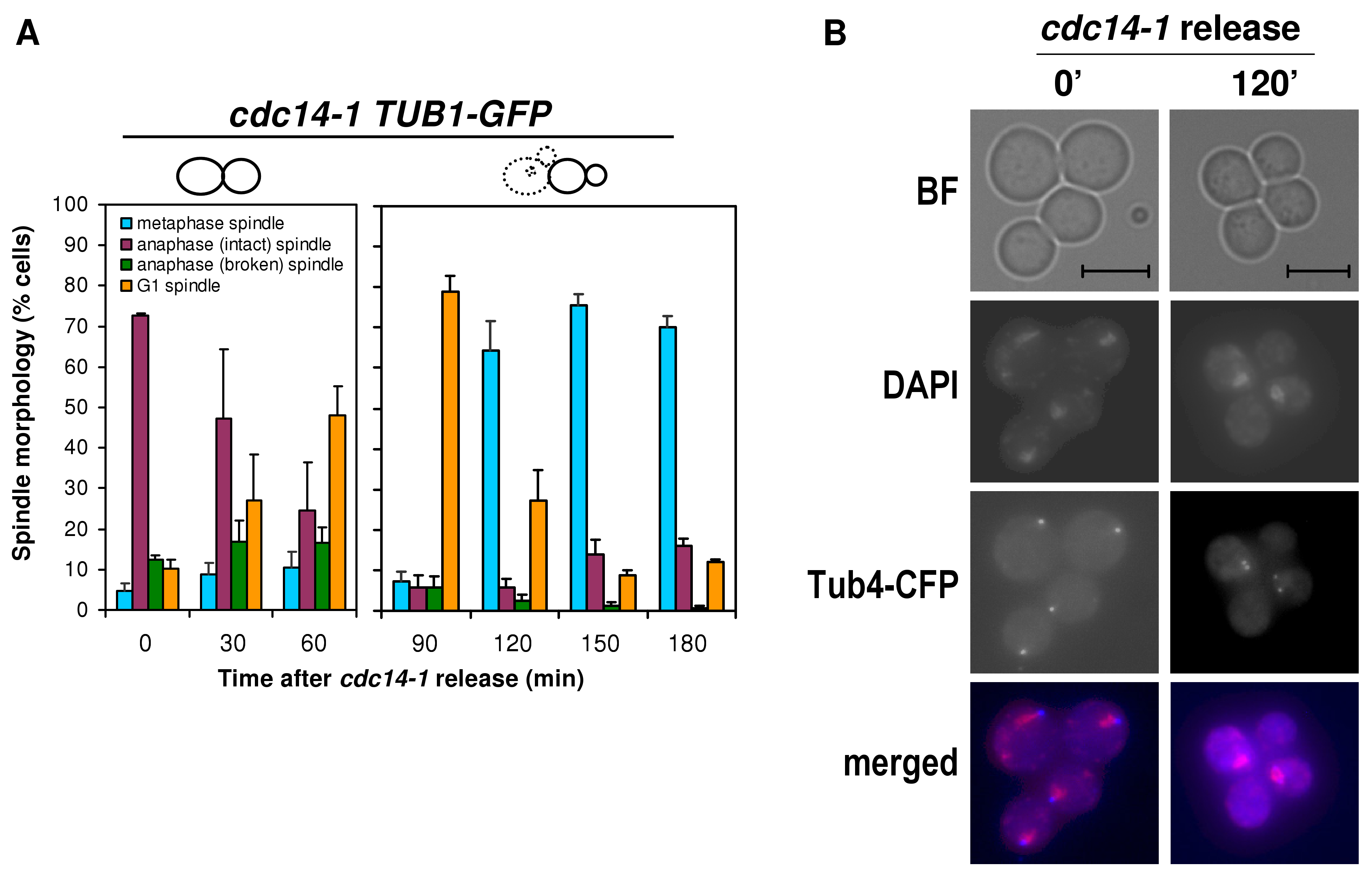

Supplement: Figure S3 — A cdc14-1 release leads to daughter cells stuck with metaphase spindles. (A) Strain FM459 (cdc14-1 TUB1-GFP) was treated as in Figure S2 and cells were scored for spindle morphology in either unbudded dumbbells (left panel) or rebudded daughter cells (right panel) (mean ± SEM, n = 3). Each rebudded daughter was counted as an individual new cell. (B) Strain FM458 (cdc14-1 TUB4-CFP) was arrested in telophase by incubation at 37°C for 3 hours (time = 0′) and then released from the arrest by dropping the temperature to 25°C. Samples taken 2 hours after the shift (120′) were stained with DAPI and analysed by microscopy. Note: Around 80% of nuclear masses have two CFP foci. Bar, 5 µm. (TIF) [file pgen.1002509.s003.tif]

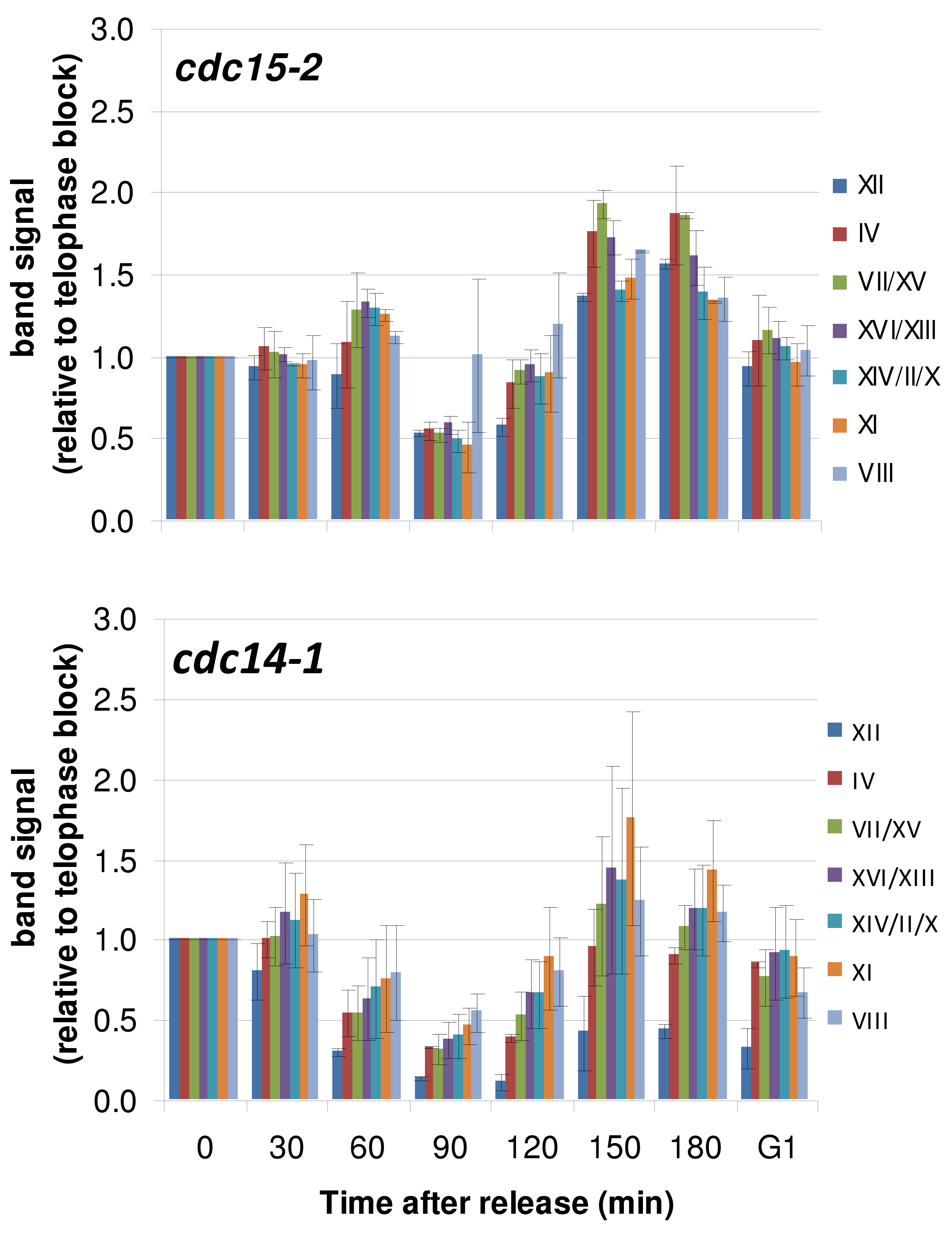

Supplement: Figure S4 — Chromosome band quantification of cdc15-2 and cdc14-1 telophase releases. The pulsed-field gel depicted in the upper panels of Figure 3B and two more independent experiments were scanned to quantify each chromosome band and normalized to that at the telophase block (mean ± SEM, n = 3). In the graphs we show the results for the two largest chromosomes (XII and IV) and for other bands containing medium size chromosomes. Note how all chromosomes entered a successful replication round (i.e., bands faded away and came back later) for both mutants; whereas chromosome XII dropped shortly after the cdc14-1 release (minute 60) and never came back in full. Also note how this drop was observed when replication was prevented by releasing into α-factor (G1 column). (TIF) [file pgen.1002509.s004.tif]

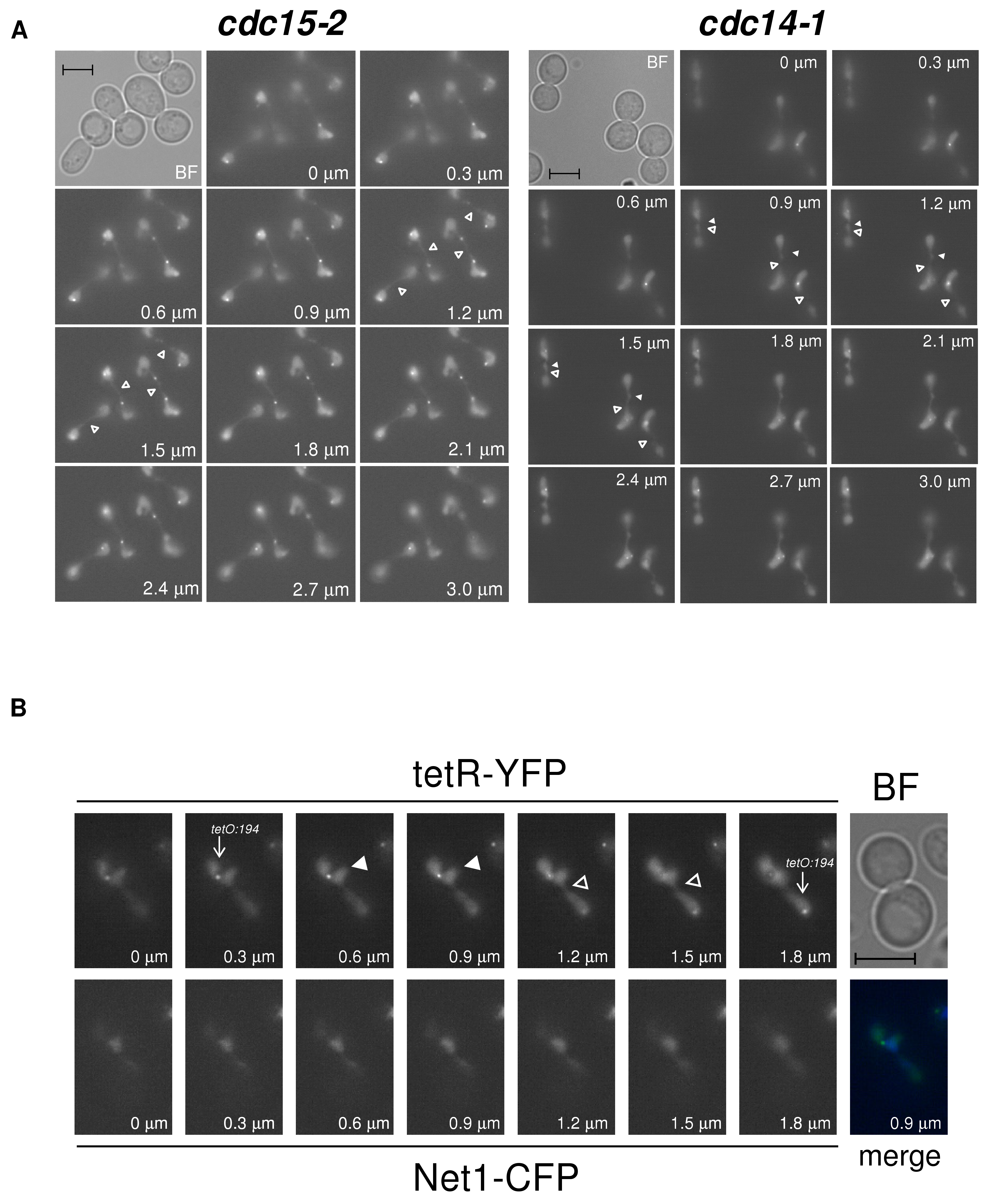

Supplement: Figure S5 — The nucleoplasm bridge of soluble TetR-YFP as seen in the cdc15-2 and cdc14-1 telophase blocks. (A) Strains FM584 (cdc15-2 tetO:487 TetR-YFP) and FM518 (cdc14-1 tetO:487 TetR-YFP) were arrested at 37°C for 3 h and micrographed. (B) Strain FM304 (cdc14-1 tetO:194 TetR-YFP NET1-CFP) was arrested as in A. Each photo represents different Z-stacks in 0.3 µm intervals. Hollow triangles point to the nucleoplasm bridge. Filled triangles point to the bulge in the bridge observed at the cdc14-1 block. Bar, 5 µm. Note how the nucleoplasm bridge is seen in all cells at both telophase blocks, the bulge is seen only in cdc14-1, and that the bulge contains the bulk of the rDNA (Net1-CFP). (TIF) [file pgen.1002509.s005.tif]

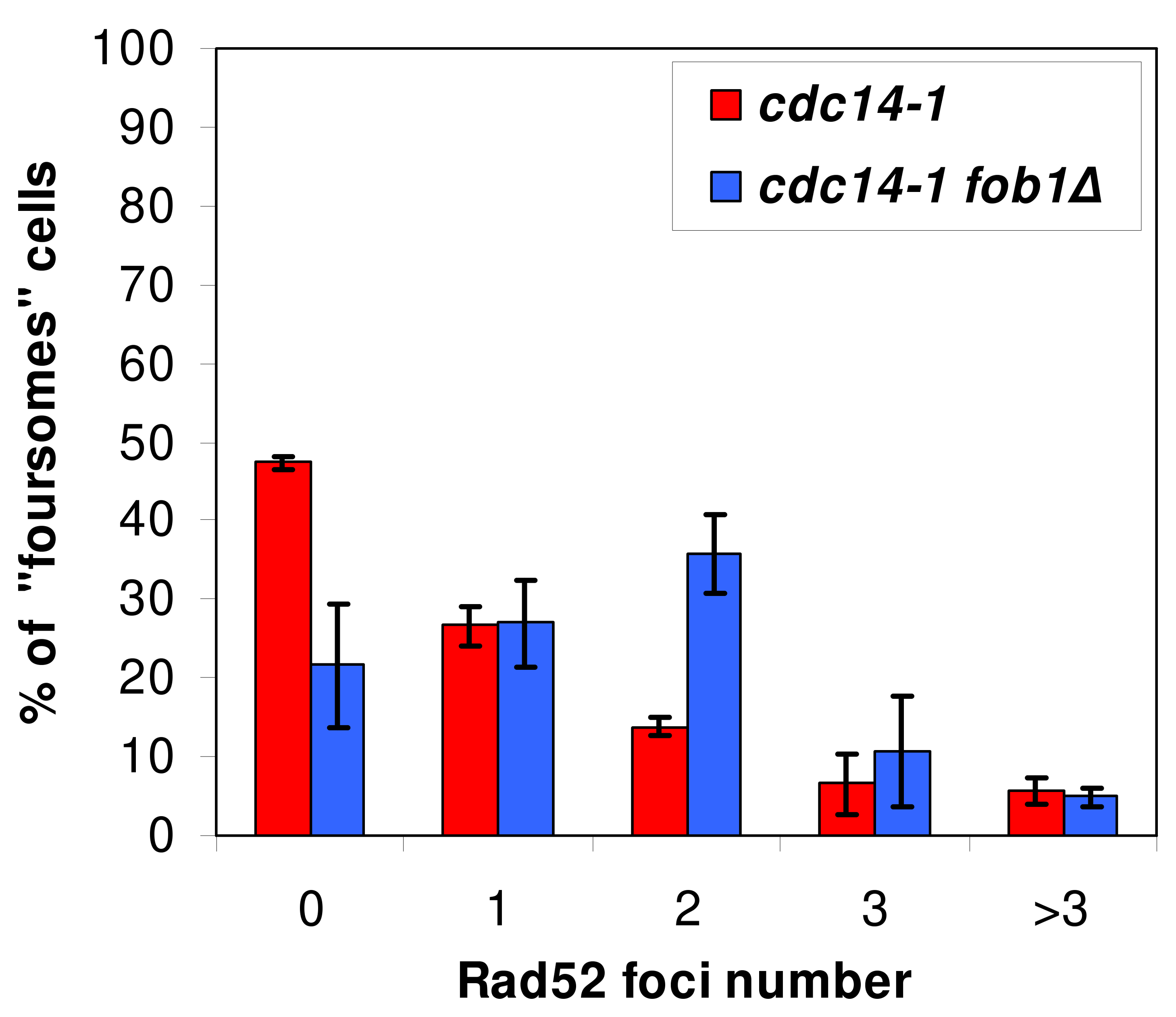

Supplement: Figure S6 — Worsening of chromosome XII segregation through deletion of FOB1 increases the number of Rad52 repair factories. Strains FM515 (cdc14-1 RAD52-YFP) and FM547 (cdc14-1 fob1Δ RAD52-YFP) were first arrested in the cdc14-1 block and then released into a new cell cycle. After 2 hours, foursomes were scored for number of Rad52 foci (mean ± SEM, n = 3). Note how foursomes with no Rad52 foci dropped from ∼50% to ∼20% when the fob1Δ mutation was present (rDNA missegregation increased from ∼50% to ∼95% relative to FOB1). (TIF) [file pgen.1002509.s006.tif]

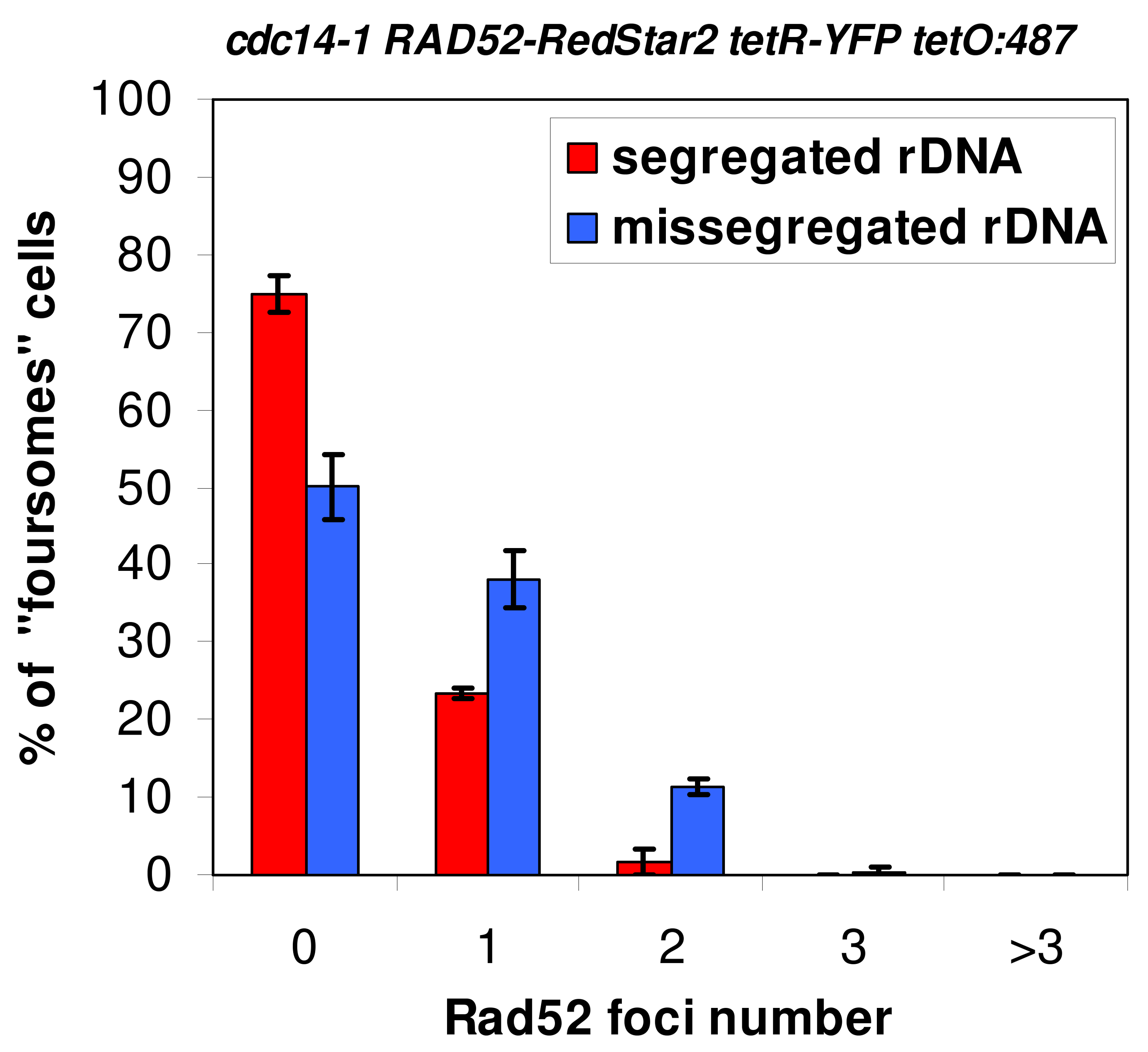

Supplement: Figure S7 — Presence of Rad52 repair factories correlates to previous failure in rDNA segregation after a cdc14-1 release. Strain FM753 (cdc14-1 RAD52-RedStar2 tetO:487 tetR-YFP) was first arrested in the cdc14-1 block and then released into a new cell cycle. After 2 hours, Rad52 foci were scored for those foursomes that have either segregated or missegregated the tetO (mean ± SEM, n = 3). (TIF) [file pgen.1002509.s007.tif]

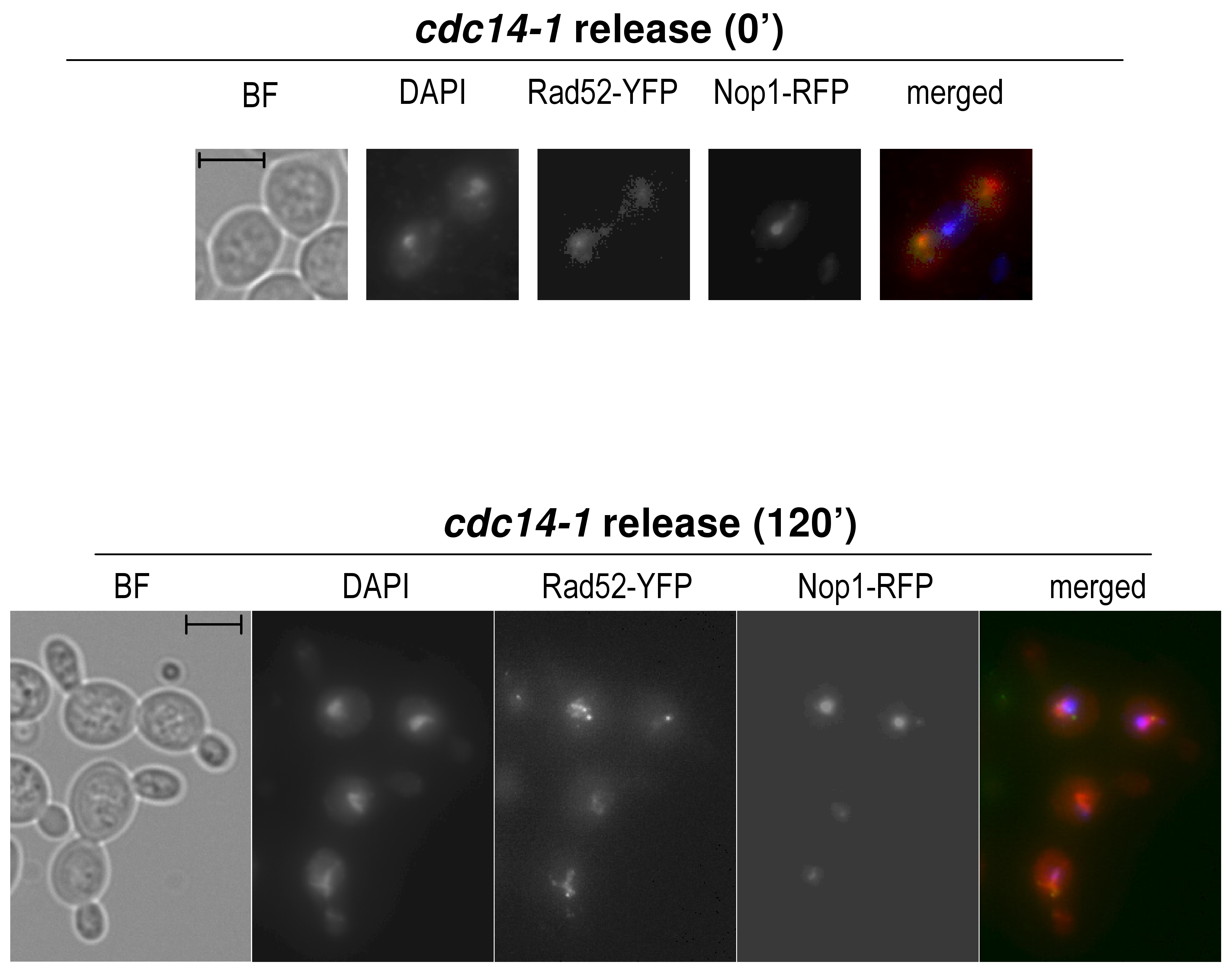

Supplement: Figure S8 — Rad52 repair factories localize out of the nucleolus after a cdc14-1 release. Strain FM460 (cdc14-1 RAD52-YFP NOP1-DsRed) was first arrested and micrographed in the telophase block (0′) and then two hours after the release (120′). Representative micrographs of the major cell types are shown. In the channel composite, DAPI is pseudocoloured in red and Nop1 in blue. Note how Rad52 foci in foursomes (at 120′) do not colocalize with the nucleolar marker Nop1. (TIF) [file pgen.1002509.s008.tif]

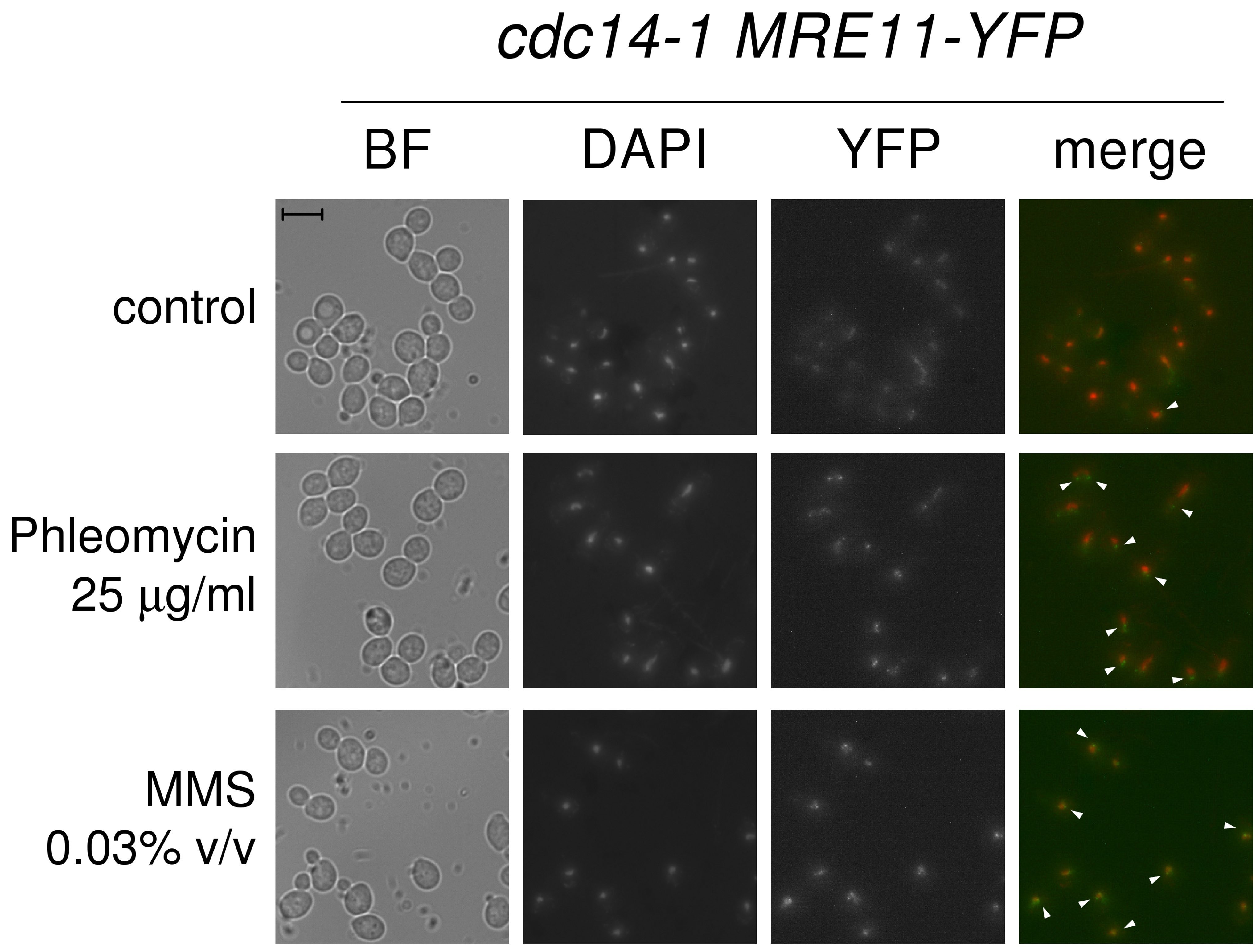

Supplement: Figure S9 — Mre11 is functional under the cdc14-1 background and concentrates in nuclear factories after chemically-generated DNA double strand breaks. Strain FM514 (cdc14-1 MRE11-YFP) was grown at 25°C until log phase and directly treated with either 25 µg/ml phleomycin or 0.03% v/v MMS. Then, samples were taken every 10 minutes and micrographed under the microscope. Mre11-YFP started concentrating in foci after just 20 minutes. Example micrographs taken after 2 hours of treatments are shown. White filled triangles point to Mre11-YFP foci. (TIF) [file pgen.1002509.s009.tif]
